# The effects of delay on objective memory and on the subjective experience of forgetting

**DOI:** 10.1038/s41598-025-26057-2

**Published:** 2025-11-25

**Authors:** Zohar Raz Groman, Talya Sadeh

**Affiliations:** 1 Department of Industrial Engineering & Management, Ben-Gurion University of the Negev, Beer Sheva, Israel; 2 Department of Psychology, Ben-Gurion University of the Negev, Beer Sheva, Israel; 3 The School of Brain Sciences and Cognition, Ben-Gurion University of the Negev, Beer Sheva, Israel

**Keywords:** Episodic memory, Contextual memories, Delay-dependent forgetting, Metacognition, Free recall, Learning and memory, Forgetting, Long-term memory

## Abstract

Recent years have seen a revived interest in the study of delay-dependent forgetting, with compelling evidence that episodic memories associated with contextual information are susceptible to delay-dependent forgetting. In addition, studies have shown that while the accessibility of memories associated with contextual information declines over time, there is little or no change in the memories’ precision and quality. Building on these findings, the current study focuses on a fundamental question regarding memories associated with contextual information, which has received scant attention: What is the subjective experience accompanying forgetting of such memories? In two experiments, we found that, in line with previous results, the accessibility of memories associated with contextual information declined following a delay, but there was little or no detrimental effect of time on the quality of the memory representations. In contrast, subjective measures of memory showed substantial decrement over time. These results are interpreted in terms of the cues that drive subjective memory ratings following a delay. Such cues include the effort of retrieval, which increases over time, even when the retrieved representation does not change (or changes only slightly), as well as subjective theories of forgetting, which may not reflect that some aspects of memory show little or no forgetting over time.

**Supplementary Information:**

The online version contains supplementary material available at 10.1038/s41598-025-26057-2.

## Introduction

For decades the cognitive literature lacked a coherent theory regarding the causes of forgetting^1^. Early research in the field assumed that memories decay over time. Later, however, the scale shifted towards interference as the primary cause of forgetting^2,3^. For years it was widely accepted that the mere passage of time does not cause degradation of memories but rather that forgetting is caused by acquiring similar information before or after learning^1^. The Representation Theory of Forgetting proposes that both interference and delay play important roles in forgetting, with the relative contribution of each depending on the nature of the cognitive representation supporting memory^4,5^. Representations of items devoid of a context, for which memory is driven by familiarity, are particularly prone to forgetting due to interference^6^. In contrast, representations of items bound to contexts, are relatively insensitive to interference from similar items. This is because such memories, even of similar items, are distinguished from one another by virtue of their association with distinct contexts. However, context decays over time, and, indeed, memories associated with contextual information are prone to delay-dependent forgetting^7,8^. In the current study, we use the term “memories associated with contextual information” to refer to memories in which a contextual element (such as a background image) plays a role in retrieval. We use the above term because the present study specifically addresses the retrieval mechanisms of such episodic memories.

Recent studies suggest a surprising pattern of delay-dependent forgetting for memories associated with contextual information: even though the probability of retrieval of such memories is reduced over time, their precision and quality remain unchanged^7,9,10^. Precision refers to the fidelity^9^ and specificity of a remembered representation—how accurately fine-grained contextual details are preserved once the memory is retrieved^9^. Precision can be operationalized as the variability of recall around the true feature value; for instance, when participants recall the spatial position of an object, higher precision is reflected by a narrower distribution of recall errors. Thus, precision captures the level of detail or resolution in the retained memory trace, rather than the probability of retrieval itself^9^.

By **“**quality”, we refer here to the integrity and richness of the memory representation — the extent to which multiple elements of the event (e.g., temporal, semantic, perceptual, and spatial context) are preserved and are bound to the item, or to one another^10,11^. Thus, memory quality reflects the *binding* between an item and its contextual features, and/or between the contextual features themselves. Evidence for preservation of memory’s quality has been demonstrated in a study examining^10,11^ retrieval of event-based memories^10^. Event-based memories were operationalized as triplets of locations (e.g., kitchen), person (e.g., Barack Obama) and objects (e.g., hammer). Memory for these triplets was examined immediately after encoding, after 12 h, and after a week’s delay. Such triplets may be regarded as a specific example of memories associated with contextual information—with one (or more) of the three elements in the triplet serving as the context. It was found that accessibility (measured as retrieval accuracy) decreased over time. However, the dependency between retrieval of pairs within triplets (e.g., kitchen-hammer and Obama-hammer) remained even after a delay. Thus, the quality of complex, event-based memories was preserved, such that the binding between contextual elements did not fragment over time.

Berens et al.^9^ also found a similar pattern of forgetting for memories associated with contextual information. In their study, they examined time-dependent changes in memory accessibility (the ability to retrieve an item) and in memory precision (e.g., how precise is memory for the location of an object). Participants learned a set of related word-location associations and were tested on them either in an immediate test or in a delayed test, 3 to 96 h after the initial study phase. The results indicate a reduction in memory accessibility over time with no change in memory precision. Thus, forgetting has been manifested in a reduction in the accessibility of the memories over time, even though their precision and/or quality remain intact.

Converging evidence for this pattern of time-dependent forgetting comes from a recent study that examined delay-dependent forgetting (immediate vs. ~15-min delay) of words superimposed on backgrounds depicting spatial contexts^7^. Memory for the words was examined using a free recall paradigm and memory for the backgrounds was probed with a multiple-choice recognition test. While the number of correctly recalled words decreased with time, given that an item was recalled, the nature of its underlying representation remained strikingly similar across both delays. This conclusion was based on a set of three findings. First, the probability of correctly remembering the background image did not differ between the two delay conditions. Second, no changes were found between conditions in the Temporal Contiguity Effect (TCE). The TCE refers to the phenomenon whereby participants tend to successively recall items from close serial positions. This effect is explained in terms of temporal context–all information peripheral to the study items (e.g., thoughts and associations) that gradually changes over time^4,12,13^. The temporal contexts of two neighbouring study items are similar, and hence, retrieval of one item is likely to trigger the retrieval of its neighbouring item that shares a similar temporal context^8^. The finding that the TCE does not vary with delay suggests that the role of temporal context in driving retrieval is not affected by delay-dependent forgetting. Third, no changes were found in semantic organization between delay conditions. Participants tended to recall words that were semantically related successively with the same probability in both delay conditions. Thus, also the role of semantic information in driving retrieval is not affected by delay-dependent forgetting. Finally, no time-dependent changes were found in organization according to background information. This was measured using background clustering—the extent to which recalling an item with a certain background triggers recall of an additional item with the same background Together, these findings demonstrate an intriguing pattern of delay-dependent forgetting, whereby with time, the accessibility of memories declines, but the qualitative nature of the recalled memories remains unchanged.

In contrast to the findings of the above study, which examined *objective* forgetting in a word-list free recall paradigms, in another study using a similar free recall paradigm it was found that the *subjective* measures of forgetting showed a different pattern^14^. The Remember/Know (R/K) procedure was used to measure the subjective experience associated with each word retrieved after a short versus longer delay (~ 20 min). Participants made subjective judgments regarding each recalled item, classifying it into one of two categories: (1) “Remember”, referring to retrieval of an item associated with contextual detail; or (2) “Know”, referring to retrieval of an item alone, which is accompanied by a feeling of familiarity. The proportion of Remember responses decreased in the longer delay. The time-dependent decline in the proportion of Remember responses is consistent with that observed in recognition studies using the R/K procedure (reviewed in Sadeh et al.^4^ and Sadeh & Pertzov^14^.

A synthesis of the evidence across the reviewed studies suggests that delay may affect only the accessibility of event-based memories or memories associated with contextual information in general, but leave the representation of those memories that are retrieved after delay unchanged. However, even though the representations of these memories remain unchanged on the objective level, they may show a decline on the subjective level (reflected in the proportion of Remember items) after a delay. Thus, it may be the case that delay-dependent forgetting exerts different effects on subjective measures of memory than it does on objective measures of memory.

Subjective measures of memory, in particular confidence, provide valuable information regarding the accuracy of one’s memory^15^. If an eyewitness says “I am absolutely confident that this is the woman I saw rob the jewellery store”, we would more likely accept their testimony as accurate than as wrong in light of their high confidence^16,17^. Thus, ample research has examined the relation between objective and subjective measures of memory^18–20^. Elucidating this relation bears great importance both on the theoretical level (e.g., are humans consciously aware of the accuracy of their memory and changes in it (or lack thereof)? ), as well as on the practical level, in uncovering the circumstances in which eyewitnesses’ subjective ratings (mostly confidence) are indicative of objective accuracy^15,17^. However, despite its importance, the subjective experience accompanying time-dependent forgetting and its relation with objective measures of forgetting currently remains a largely unexplored area of study.

The few studies that explored the objective-subjective relationship after a delay reported inconsistent results. Spearing and Wade^21^ investigated the effect of retention interval on the relationship between eyewitness memory accuracy and confidence for a mock crime video with a cued-recall test either immediately following encoding, after 1 week, or after 1 month. Their results showed that a longer delay between encoding and retrieval phases (immediate/ 1-week / 1-month) led to lower objective accuracy. However, it also led to a decrease in objective-subjective calibration, which was manifested in the immediate test as slight over-confidence and in the 1-week and 1-month delay tests as under confidence. The under confidence was driven by the relatively high number of correct responses at the low confidence levels in the two long delay conditions. Hence, participants adjust their confidence to reflect their memory accuracy after a long retention interval, even if this adjustment does not strengthen the confidence-accuracy relationship. Odinot and Wolters^22^ also showed an effect of retention interval on objective-subjective relation. In their study, the influence of retention interval on the accuracy and confidence measures of episodic memory was investigated by testing participants’ cued recall performance for a movie in 3 different retention intervals: a delay of a 1-week delay, three weeks, and five weeks. Each participant was tested three times with the same questions (some were specific questions like “what was the woman wearing?” and some were more general like “give a full description of the sitting room”). The results showed that there was no difference in the level of accuracy between the consistent details and the inconsistent details (whether the detail was remembered at the previous retention intervals). In contrast, there was a significant difference in confidence, with consistent details receiving higher confidence ratings than inconsistent details. Finally, Sekeres et al.^23^ examined the forgetting of short video clips over time (0, 1, 3, and 7 days). Objective measures of memory performance were obtained by scoring participants’ verbal recollections of the film clips. Subjective measures were obtained by having participants rate their confidence in memory for the story content and the vividness of the details. It was found that the subjective ratings decreased over time, even when the objective measures of memory remained unchanged (because no forgetting was observed). Hence, a discrepancy between objective and subjective memory performance was observed. However, this was the case when no objective forgetting occurred.

The aim of the current study was to investigate how delay-dependent forgetting affects subjective measures (i.e., confidence ratings) of memory, specifically for memories associated with contextual information, whose representations’ quality has been shown relatively stable over time. Hence, on the objective level, these memories are thought to show a decline in accessibility over time, with little or no change in their underlying representations [Our hypotheses were inspired by the literature on event-based memory, where ‘event’ is defined as an association between a person, location and object. Still, our focus here is not specifically on such event-based memories, but rather on memories associated with contextual information (e.g., an item presented on a background image)]. We aimed to explore the subjective experience of time-dependent forgetting, which manifests as a decline in accessibility but (relative) stability in the underlying memory representations. We hypothesized that subjective measures of memories would decline over time, even for items that are correctly retrieved and whose representations’ quality remains relatively stable.

This study contains two experiments with two delay conditions each: a Short-delay and a Long-delay. In Experiment 1, word lists were utilized, which were replaced by line-drawing objects in Experiment 2. This modification was made due to the relatively large number of participants in Experiment 1 that had to be excluded because they produced no correct recalls in the Long-delay condition. The choice of line drawings was based on results from a previous study, which showed that over 30% of 50 studied line drawings were recalled after a week’s delay^24^. Both of our experiments employed two lists of items that appeared on a background of either a sea or a forest image. The Short-delay condition included a distraction task followed by an immediate free recall test, while the Long-delay condition involved recall after 24 h. Confidence ratings were collected after each recall. The confidence rating scale was modified from a scale of 1 to 6 to a scale of 0% to 100% in Experiment 2 to enhance sensitivity and to make it more intuitive. One of the main motivations for conducting Experiment 2 was to directly assess the accessibility of memory traces through reaction times (RTs), which were not measured in Experiment 1. RTs are a valid measure of retrieval fluency^25,26^, which in turn provides an objective means of the cognitive effort involved in recall. In We assessed retrieval fluency through inter-response times (IRTs)— the time between consecutive recalls. We hypothesized that individuals’ confidence judgments are partially experience-based—namely, affected by retrieval fluency or the ease with which an item comes to mind^25^, and that retrieval fluency is modulated by delay^27–29^.

### Study aims

First, we expected to replicate previous results regarding the qualitatively-intact nature of memories associated with contextual information after delay. Thus, we expected an overall decrease in the number of correctly recalled words with time^7^, but no change in the nature of the underlying representation of each event (i.e., word)—as operationalized by recall organization factors (Temporal organization, Semantic organization, and memory background clustering). Second, and most importantly, we predicted a discrepancy in the effects of delay on objective and subjective measures of memory. Thus, even though the objective quality of memory was expected to be similar in both conditions, the subjective measure (namely, the confidence ratings) was expected to be lower in the Long-delay condition than in the Short-delay condition.

In summary, converging evidence suggests that delay-dependent forgetting primarily affects the accessibility of contextual memories, while the precision and quality of the retained representations remain relatively stable. However, the subjective experience that accompanies this form of forgetting —how individuals experience their memory after a delay —has received little empirical attention. The present study was therefore designed to address this lacuna by examining how delay influences both objective and subjective aspects of memory performance for representations whose quality remains intact. This approach allows us to determine whether subjective aspects of memory (i.e., confidence) decline over time, even when the underlying memory representations remain qualitatively intact.

## Experiment 1

### Results

Descriptive Statistics for all Experiment 1 variables are presented in the Appendix (Table S1).

#### Objective measures

##### Recall rate and accuracy

Participants recalled an average of *8.46* words (SD = 2.67) in the Short-delay condition and 4.96 words (SD = 2.39) in the Long-delay condition. Of these, the proportion of correctly recalled words was significantly higher in the Short-delay condition (M = 0.94, SD = 0.11) compared to the Long-delay condition (M = 0.88, SD = 0.18; t(75) = 2.15, Cohen’s d = 0.25, *p* = .03, 95% CI [0.004, 0.10], BF_10_ = 1.10).

##### Source discrimination

Six participants were excluded from this analysis because did not correctly remember any background in the Long-delay condition. The proportion of correct source discrimination out of correctly recalled words was higher in the Short-delay condition (M = 0.82, SD = 0.19) than in the Long-delay condition (M = 0.68, SD = 0.24; t(69) = 4.43, Cohen’s d = 0.53, *p* < .001, 95% CI [0.08, 0.20], BF_10_ = 564.52).

##### Recall organization for correctly recalled words

**Fig. 1:**
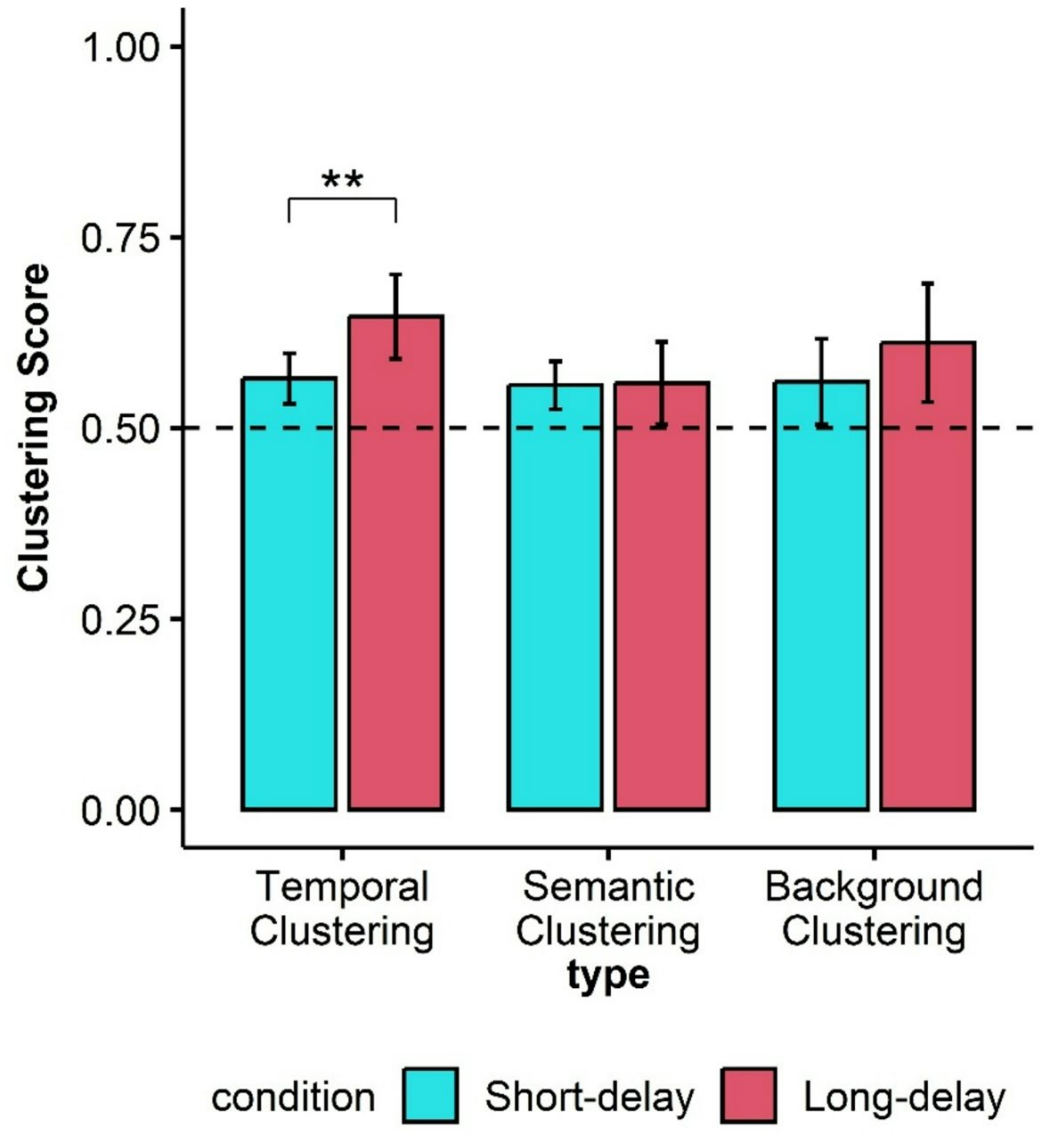
Experiment 1 Clustering scores per condition. A dashed horizontal line indicates chance-level recall organization. Error bars represent 95% confidence intervals around the mean. ** < 0.01.

Recall organization data are presented in Fig. 1. To calculate the temporal factor and the background clustering for a participant’s recall in a given condition, there must be at least two consecutive correct recalls. These factors cannot be computed if this condition is not met at any point in the recall sequence. The semantic factor calculation requires at least two consecutive recalls, where each word is either correct or a word with available semantic information. The mean Temporal Factor scores for both delay conditions were greater than chance (0.5). For the Short-delay condition, the Temporal Factor score was 0.56 (SD = 0.14, t(75) = 3.88, Cohen’s d = 0.45, *p* < .001, BF_10_ = 97.73), and for the Long-delay condition, the Temporal Factor score was 0.65 (SD = 0.24, t(75) = 5.25, Cohen’s d = 0.60, *p* < .001, BF_10_ = 11,237). A paired-sample t-test found a significant difference between delay conditions (t(75) = -2.76, Cohen’s d = -0.32, *p* = .007, 95% CI [-0.14, -0.02], BF_10_ = 4.23). Thus, temporal organization does not decline with time, and actually improves.

The mean Semantic Factor scores were above chance (0.5) for both delay conditions. For the Short-delay condition, the Semantic Factor score was 0.56 (SD = 0.14, t(75) = 3.56, Cohen’s d = 0.41, *p* < .001, BF_10_ = 36.99); for the Long-delay condition, the mean Semantic Factor score was 0.56 (SD = 0.24, t(75) = 3.56, Cohen’s d = 0.41, *p* < .001, BF_10_ = 36.99). No differences were found between the two delay conditions (t(75) = -0.08, Cohen’s d = -0.18, *p* = .19, 95% CI [-0.06, 0.06], BF_01_ = 7.90).

Next, we examined whether background pictures influenced recall organization through background clustering. This measure gives an estimate of the degree to which two items sharing a similar background at encoding cluster together at recall. Hence, it is a measure of implicit retrieval of the background of an item. The mean of Background-Clustering scores was greater than chance in the Short-delay condition (M = 0.56, SD = 0.25, t(75) = 2.14, Cohen’s d = 0.25, *p* = .02, BF_10_ = 1.07), and in the Long-delay condition (M = 0.61, SD = 0.34, t(75) = 2.86, Cohen’s d = 0.33, *p* = .003, BF_10_ = 5.42). The difference between conditions was not significant (t(75) = -1.05, Cohen’s d = -0.12, *p* = .29, 95% CI [-0.15, 0.04], BF_01_ = 0.93). The clustering results indicate that recall organization—whether temporal, semantic, or background—was not negatively affected by delay.

#### Errors data

##### Intrusions recall

Errors in free recall fall into two primary categories: prior-list intrusions (PLIs) and extra-list intrusions (ELIs). PLIs occur when participants mistakenly recall words from previous lists rather than the most recent one, contrary to instructions. ELIs, on the other hand, involve recalling words that were never presented at any point in the experiment. For the current study analyses, and due to a small number of ELIs and PLIs in each condition, we combined them into one variable called “Intrusions recalls”. In the Short-delay condition, the mean number of intrusions recall was 0.45 (SD = 0.84); in the Long-delay condition, it was 0.68 (SD = 1.24) with no significant difference between delay conditions (t(75) = -1.25, Cohen’s d = -0.14, *p* = .22, 95% CI [-0.55, 0.12], BF_01_ = 3.77).

##### “Don’t remember” selected background

The results show that there is a significant difference between the proportions of the ‘Don’t remember’ selection in the Short-delay condition (M = 0.13, SD = 0.22) and in the Long-delay one (M = 0.35, SD = 0.55), (t(75) = -3.39, Cohen’s d = -0.39, *p* = .002, 95% CI [-0.36, -0.09], BF_10_ = 22.37).

#### The subjective experience of forgetting

**Fig. 2:**
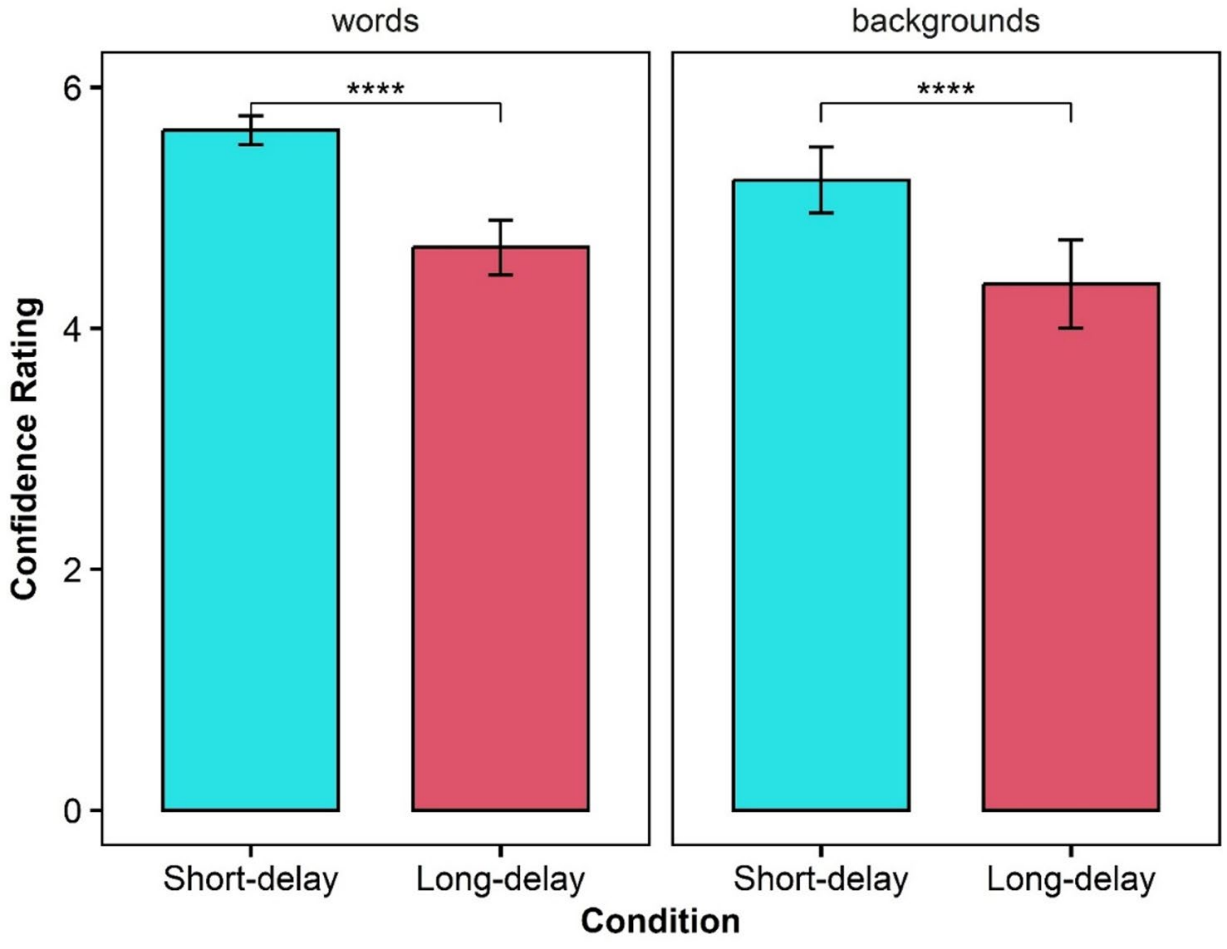
Confidence rating of words and backgrounds in each delay condition.

**Fig. 3:**
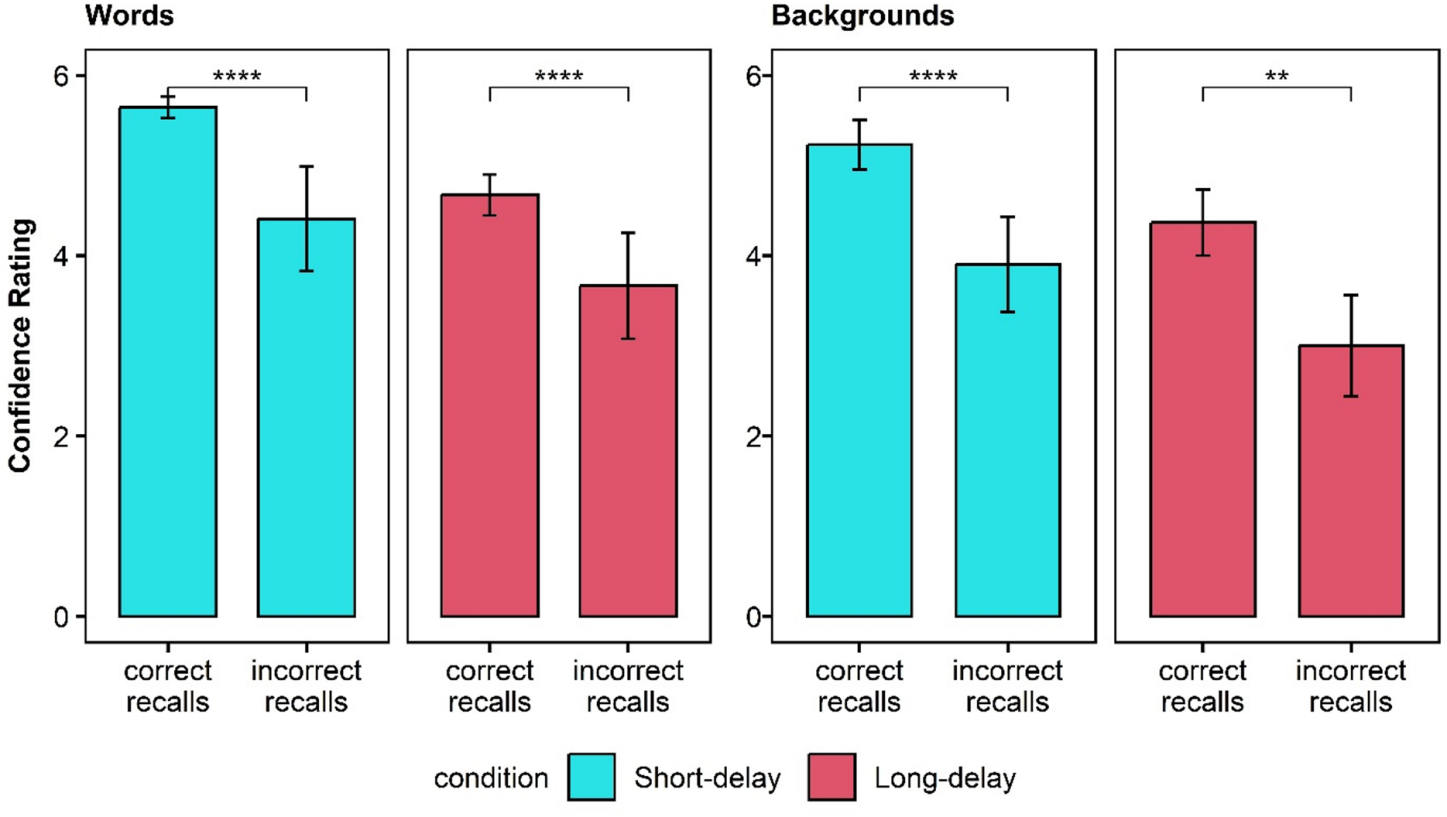
Experiment 1 confidence rating for intrusions and correctly recalled for words and backgrounds for each delay condition.

We measured participants’ ability to subjectively distinguish between accurate and inaccurate trials^30^. Delay may have a negative effect on this ability, such that items that are harder to retrieve will be associated with lower confidence ratings, even when the quality of their mnemonic representations is resistant to delay. Table 1 presents the means of confidence ratings for intrusions and correctly recalled words, as well as for backgrounds memory, per condition. Figure 2 presents the confidence ratings of words and backgrounds in each delay condition, and Fig. 3 presents the confidence rating data for intrusions and correctly recalled words and for backgrounds memory per condition.

In this analysis, we investigated how the accuracy of responses (correct recall vs. intrusions recall) and experimental conditions (immediate vs. delay) influence participants’ confidence ratings. The hierarchical structure of the data, with repeated measures nested within subjects, required using a mixed-effects model to account for individual variability. Given the limited sample size, a Bayesian approach was adopted. This method allows for the inclusion of weakly informative priors, such as a normal prior on the fixed effects, which stabilizes parameter estimates and reduces overfitting in datasets with low observation counts. The model incorporates random intercepts and slopes for accuracy and condition at the subject level, capturing individual baseline differences in confidence and variability in the effects of accuracy and condition. Using this approach, we balanced the model’s complexity with the dataset’s constraints, ensuring robust, interpretable inferences.

The Bayesian mixed-effects analysis of confidence ratings revealed clear main effects of both accuracy and delay. Specifically, participants reported higher confidence for correct recalls compared to intrusions (95% HDI [0.95, 1.82]) and lower confidence in the Long-delay condition compared to the Short-delay condition (95% HDI [− 1.37, − 0.14]). The interaction between accuracy and delay was inconclusive, as the 95% HDI interval included zero (95% HDI [− 0.82, 0.39]), suggesting that the accuracy-related confidence advantage was largely consistent across delay conditions. Importantly, the random-effects estimates indicated meaningful inter-individual variability in baseline confidence levels, as well as in the magnitude of the accuracy and delay effects, underscoring that participants differed not only in their general confidence but also in how strongly their ratings were shaped by response accuracy and memory delay.

For source discrimination, the Bayesian mixed-effects model indicated substantial evidence for effects of both condition and accuracy. Here too, the six participants who did not correctly remember any background in the Long-delay condition were excluded from the analysis. Confidence was higher for correct than for incorrect background memory (95% HDI [0.93, 1.61]) and higher in the Short-delay condition compared to the Long-delay condition (95% HDI [− 1.26, − 0.27]). A simple effects analysis indicated that in both delay conditions, participants were more confident in correct than in incorrect background memory: In the Short-delay condition, the difference was about 1.30 (95% HDI [0.90, 1.70]), and in the Long-delay condition the difference was about 1.00 (95% HDI [0.60, 1.40]). Confidence in correct background memory was also higher in the Short-delay than in the Long-delay condition (difference ≈ 0.90, 95% HDI [0.40, 1.20]). The interaction between accuracy and delay was inconclusive, as the 95% HDI included zero (95% HDI [− 0.63, 0.43]), suggesting that the confidence advantage for correct recalls was relatively stable across delay conditions. Random-effects estimates indicated substantial inter-individual variability in baseline confidence (intercept variance 95% HDI [0.48, 1.67]) and in the delay effect (95% HDI [0.19, 1.67]); variability in the accuracy effect was less certain (95% HDI [0.00, 0.79]). The model showed good overall fit, with a median Bayesian R² of approximately 0.73, indicating that a large proportion of variance in background confidence ratings was explained.

**Table 1:** Experiment 1. Results of the subjective measures of memory.

		Short-delay		Long-delay	
		M	SD	M	SD
Correct recall	Confidence for words	5.65	0.53	4.67	0.99
	Confidence for backgrounds	5.23	1.15	4.37	1.54
Intrusions recall	Confidence for words	4.41	1.41	3.66	1.58
	Confidence for backgrounds	3,90	1.57	3	1.58

## Discussion

Results of this experiment generally replicated previous results showing that the nature of the underlying representation of correctly-retrieved memories associated with contextual information is not, or only slightly, affected by time-dependent forgetting^7^. Our results show a decline in accessibility to the information^31,32^, as fewer items were correctly recalled in the Long-delay condition compared to the Short-delay condition. In contrast to previous results^7^, however, we found a significant difference in the accuracy of source discrimination between conditions.

Regarding memory organization, our results showed no significant time-dependent reductions in semantic or background clustering, which aligns with previous results^9,10,33^. In fact, our results indicate an increase in temporal organization over time. Taken together, our results suggest that while memory access becomes harder with increasing delay, the nature of the underlying memory representation remains relatively stable. With regard to the subjective measures of memory, we found that even for correctly recalled items, whose mnemonic representations remained relatively stable over time, there was a substantial decline in confidence. Despite the stability of the underlying memory representations, this decline in subjective confidence can partly be explained by a reduction in retrieval strength over time^34^. As delay increases, retrieval strength diminishes, leading to less confident retrieval. This change in retrieval strength explains why subjective confidence tends to decline, even when the underlying memory remains intact. Hence, after a delay, there is a discrepancy between the objective quality of the memory and participants’ subjective assessment of their memory.

The confidence ratings for correctly retrieved words and backgrounds in the Short-delay condition were very high, often approaching ceiling levels. While this may indicate a potential ceiling effect, it does not impact the primary results under discussion. We found that participants could subjectively distinguish between accurate and inaccurate words and backgrounds relatively well in Short and Long-delay conditions. However, the gap in confidence ratings between intrusions and correctly recalled words was influenced by condition; for words, the gap was larger in the Long-delay condition, whereas for backgrounds, the gap was larger in the Short-delay condition. This pattern underscores that although there is a ceiling effect in the Short-delay condition, there is no such ceiling effect in the Long-delay one. Therefore, the two conditions differ at least in this regard.

The error results indicate that participants placed the search criterion for retrieving an item in a lower position in the Long-delay condition than in the Short-delay condition^20^. The search criterion describes the cognitive strategy used to retrieve information, which is influenced by task demands and individual expectations about memory accuracy^35^. Thus, in the Short-delay condition, participants mostly retrieved items with high confidence and minimal mistakes. In contrast, participants made more mistakes in the Long-delay condition and showed more significant variability in confidence ratings. In other words, participants retrieved words and remembered backgrounds they were not very confident of, and hence, made more mistakes in Long-delay conditions.

## Experiment 2

### Results

Descriptive Statistics for all Experiment 2 variables are presented in the Appendix (Table S2).

#### Objective memory performance

##### Recall rate and accuracy

Participants recalled an average of 13.5 objects (SD = 7.02) in the Short-delay condition and 9.01 objects (SD = 5.10) in the Long-delay condition. Of these, the proportion of correctly recalled objects was significantly higher in the Short-delay condition (M = 0.87, SD = 0.11) compared to the Long-delay condition (M = 0.77, SD = 0.16), as in Experiment 1. A paired-sample t-test confirmed the statistical significance of this result (t(86) = 6.62, Cohen’s d = 0.71, *p* < .001, 95% CI [0.07, 0.13], BF_10_ = 3,700,783).

##### Source discrimination

Six participants were excluded from all source discrimination analyses because they did not correctly remember any background in the Long-delay condition. The proportion of correct source discrimination for correctly recalled objects was significantly higher in the Short-delay condition (M = 0.7, SD = 0.18) than in the Long one (M = 0.62, SD = 0.2) (t(80) = 3.52, Cohen’s d = 0.39, *p* < .001, 95% CI [0.03, 0.12], BF_10_ = 32.98).

##### Recall organization for correctly recalled objects

**Fig. 4:**
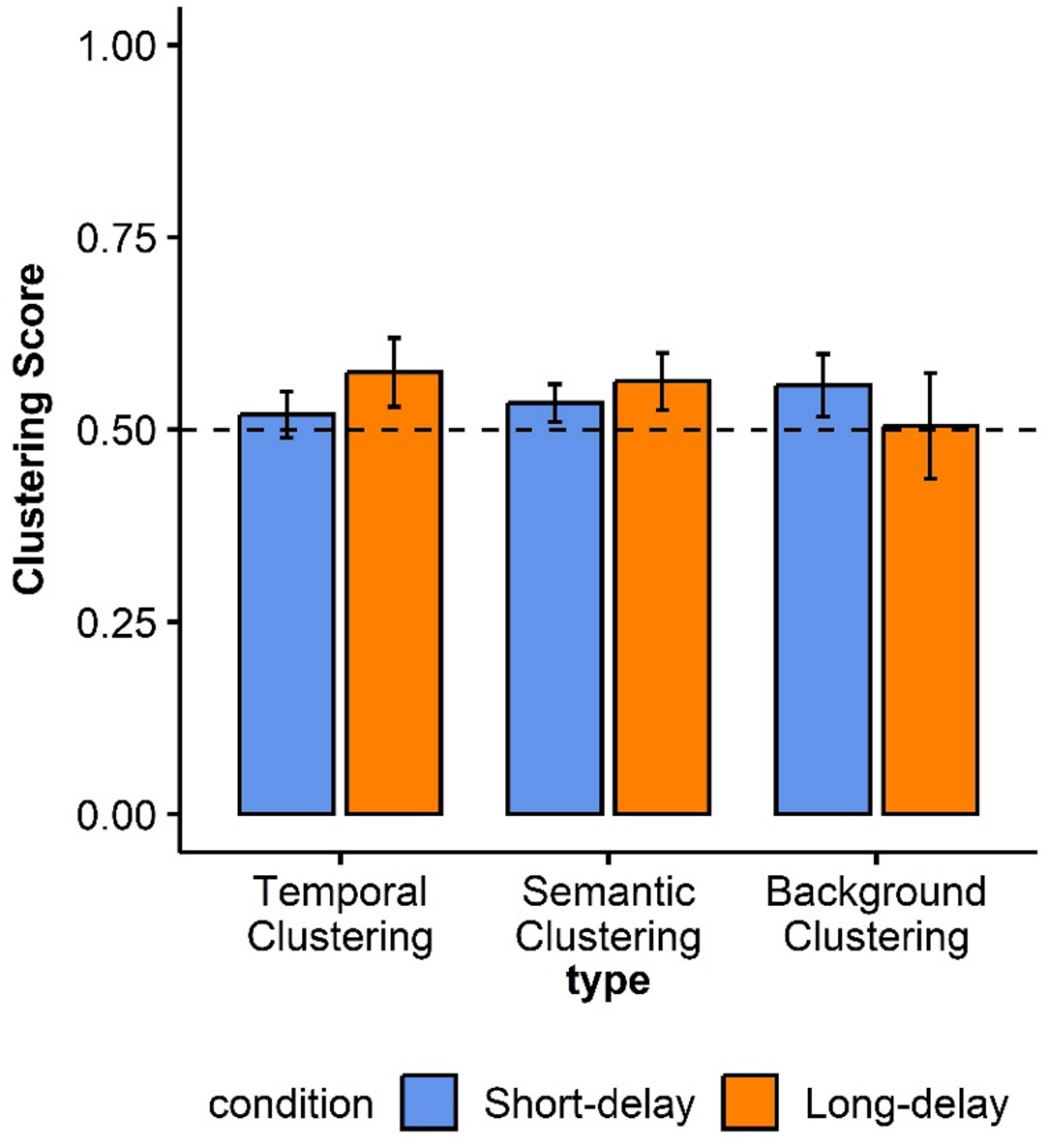
Experiment 1 Clustering scores per condition. A dashed horizontal line indicates chance-level recall organization. Error bars represent 95% confidence intervals around the mean.

Recall organization data are presented in Fig. 4. The means of the Temporal Factor scores were greater than chance (0.5) for the Long-delay condition, but not for the Short-delay one. For the Short-delay condition, the Temporal Factor score was 0.52 (SD = 0.14, t(86) = 1.29, Cohen’s d = 0.14, *p* = .09, BF_01_ = 3.78), and for the Long-delay condition, the Temporal Factor score was 0.57 (SD = 0.21, t(86) = 3.32, Cohen’s d = 0.36, *p* < .001, BF_10_ = 18.03). A marginally significant difference was found between delay conditions for temporal clustering (t(86) = -1.98, Cohen’s d = -0.21, *p* = .051, 95% CI [-0.11, 0.00], BF_01_ = 1.31).

For Semantic organization, the means of semantic clustering scores for both delay conditions were also greater than chance (0.5). For the Short-delay condition, the Semantic Factor score was 0.53 (SD = 0.11, t(86) = 2.81, Cohen’s d = 0.30, *p* = .003, BF_10_ = 4.66), and for the Long-delay condition, the Semantic Factor score was 0.56 (SD = 0.17, t(86) = 3.34, Cohen’s d = 0.36, *p* = .001, BF_10_ = 19.41). No statistically significant differences between delay conditions were found (t(86) = -1.28, Cohen’s d = -0.14, *p* = .20, 95% CI [-0.07, 0.01], BF_01_ = 3.83).

Last, the means of the background clustering scores were greater than chance in the Short-delay condition (M = 0.56, SD = 0.19, t(86) = 2.78, Cohen’s d = 0.30, *p* = .003, BF_10_ = 4.27), but not in the Long-delay condition (M = 0.51, SD = 0.32, t(86) = 0.13, Cohen’s d = 0.01, *p* = .44, BF_01_ = 8.38). No statistically significant differences were found between delay conditions (t(86) = 1.36, Cohen’s d = 0.15, *p* = .18, 95% CI [-0.02, 0.13], BF_01_ = 3.47).

#### Errors data

##### Intrusions recall

In the Short-delay condition, the mean number of intrusions recall was 1.68 (SD = 1.57); in the Long-delay condition, it was 2.12 (SD = 1.76) with a significant difference between delay conditions (t(86) = -2.66, Cohen’s d = − 0.0.29, *p* =. 009, 95% CI [-0.76, -0.11], BF_10_ = 3.22).

##### “Don’t remember” selected background

The results show a significant difference between the ‘Don’t remember’ selection proportions in the Short-delay condition (M = 0.23, SD = 0.20) versus the Long-delay one (M = 0.46, SD = 0.41). (t(86) = -5.69, Cohen’s d = -0.61, *p* < .001, 95% CI [-0.30, -0.15], BF_10_ = 75,189).

#### Subjective experience of forgetting

**Fig. 5:**
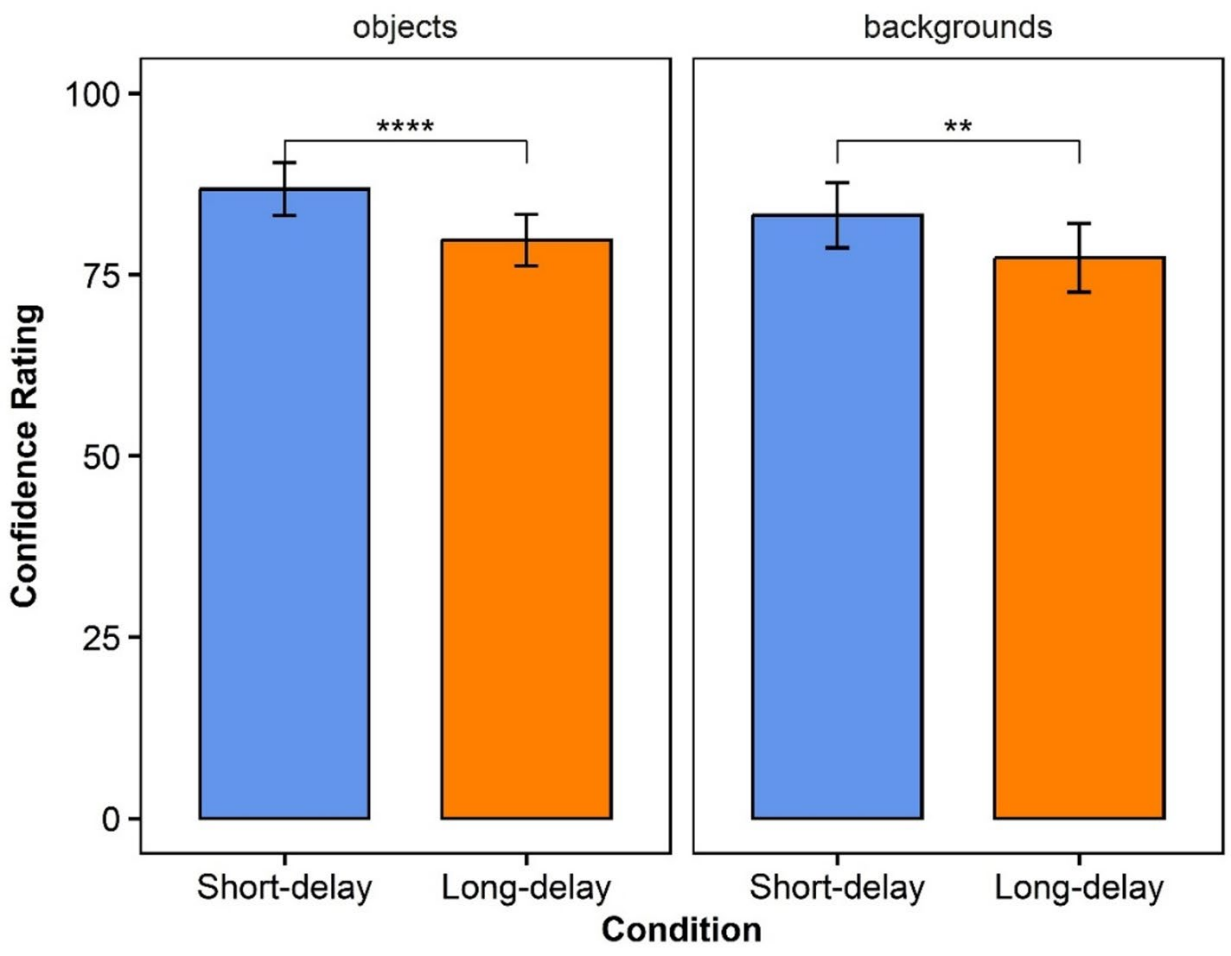
Confidence rating of objects and backgrounds in each delay condition.

**Fig. 6:**
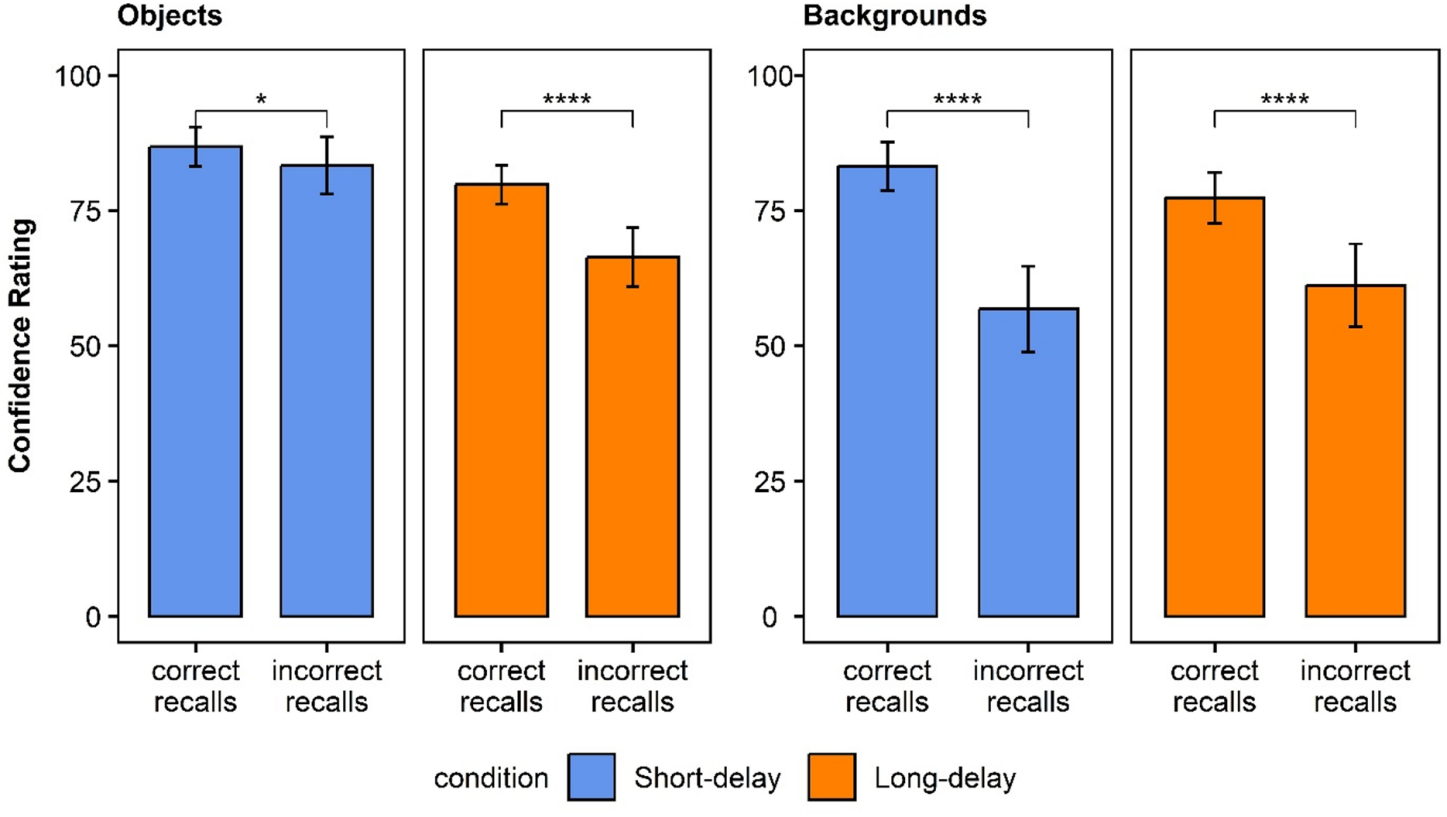
Experiment 2 Confidence rating for intrusions and correctly recalled objects for objects and backgrounds for each delay condition.

Table 2 presents the means of confidence ratings for intrusions and correctly recalled objects and backgrounds per condition. Figure 5 presents the confidence ratings of objects and backgrounds between delay conditions, and Fig. 6 presents the confidence rating data for intrusions and correctly recalled objects and backgrounds.

Consistent with the results of Experiment 1, the Bayesian mixed-effects analysis revealed a main effect of accuracy, with higher confidence ratings for correct compared to intrusion recalls (median difference = 3.90, 95% Highest Posterior Density (HPD) interval [0.49, 7.11]). A main effect of condition also emerged, with higher confidence in the Short-delay than the Long-delay condition (difference = 5.76, 95% HPD [2.49, 8.88]). Post-hoc comparisons confirmed these effects: In the Short-delay condition, participants were more confident in correct than in intrusion recalls (difference = 3.90, 95% HPD [0.49, 7.11]), and this pattern was even more pronounced in the Long-delay condition (difference = 11.17, 95% HPD [6.98, 15.29]). Confidence for correct recalls was also significantly higher in the Short-delay than in the Long-delay condition (difference = 5.76, 95% HPD [2.49, 8.88]). The interaction between accuracy and delay revealed substantial evidence for a modulation of confidence: the drop in confidence from correct to intrusion recalls was more pronounced in the Long-delay condition (interaction estimate = − 7.27, 95% HPD [− 10.9, − 3.52]).

Random-effects estimates indicated substantial inter-individual variability in baseline confidence (intercept variance credible interval well above zero), as well as in the effects of accuracy and delay. This variability suggests that while the general pattern of higher confidence for correct recalls and reduced confidence after a delay was consistent, the magnitude of these effects differed considerably across participants. The model demonstrated good explanatory power, with a median Bayesian R² of approximately 0.54, suggesting that a moderate-to-large proportion of variance in confidence ratings was accounted for.

The Bayesian mixed-effects model for background discrimination similarly revealed robust effects of both recall accuracy and delay. In this analysis. The six participants who did not remember any correct background in the Long-delay condition were excluded. Confidence ratings were higher for correct than for incorrect background memory (95% HPD [5.00, 13.49]). The effect of delay was not reliably supported, with the 95% HPD for the Short–Long contrast including zero (95% HPD [− 6.58, 0.89]). The accuracy × delay interaction was likewise inconclusive (95% HPD [− 4.92, 2.32]), suggesting that the confidence advantage for correct background memory was broadly stable across delay conditions.

Random-effects estimates indicated pronounced inter-individual variability in baseline confidence and in the size of the experimental effects. Specifically, the variance of the random intercept was large (95% HPD [356.42, 938.09]), as were the variances of the accuracy effect (95% HPD [202.13, 735.15]) and the delay effect (95% HPD [196.56, 780.67]). Moreover, intercept–effect covariances were credibly negative (intercept–accuracy 95% HPD [− 583.31, − 77.52]; intercept–delay 95% HPD [− 555.16, − 105.05]), implying that participants with higher baseline confidence tended to show smaller accuracy and delay effects. Taken together, the model captured a moderate-to-large proportion of variance (median Bayesian R² ≈ 0.55), revealing robust accuracy-related differences in confidence alongside substantial heterogeneity across individuals.

**Table 2:** Experiment 2. Results of the subjective measures of memory.

		Short-delay		Long-delay	
		M	SD	M	SD
Correct recall	Confidence for objects	86.8	17.1	79.8	16.7
	Confidence for backgrounds	83.2	20.3	77.3	21.4
Intrusions recall	Confidence for objects	833	22.3	66.4	23.7
	Confidence for backgrounds	56.8	29.7	61.2	25.7

##### Inter-response times between two consecutive recalls

As standard practice in RT analysis, we used a filter to remove IRT outliers, observations of 3 standard deviations above or below each participant’s mean score. IRTs below 100ms were also removed from the data. Notably, no observations were excluded from our dataset based on these criteria. First, a linear mixed-effects model was conducted to examine the effects of condition (Short vs. Long-delay) and accuracy (Correct recall vs. Intrusions recall) on recall times. The results revealed a significant main effect of condition F(1, 129.18) = 50.99, η² = 0.28, *p* < .001), indicating inter-response times were shorter in the Short-delay condition than in the Long-delay. The main effect of recall accuracy was marginally significant (F(1, 122.46) = 3,84, η² = 0.03, *p* = .052), suggesting a trend toward longer recall times for incorrect (intrusion) responses compared to correct recalls. However, the interaction between condition and recall accuracy was insignificant (F(1, 243.04) = 0.08, η² < 0.001, *p* = .78).

Next, we examined the relation between IRTs and confidence ratings. We calculated the correlation between IRTs and confidence ratings for correct answers for each participant in each delay condition separately. The averages across all participants, for each delay condition, were computed and compared to 0 and to one another. To this end, we transformed the correlation coefficients using the Fisher transformation (the confidence intervals’ data are reported in non-transformed correlation coefficients to enhance readability). Sixteen participants were excluded from the analysis because, after restricting the data to correct recalls and applying the standard RT trimming procedure, their remaining data exhibited no variance in confidence ratings (e.g., all items had the same confidence value or only a single item remained), precluding correlation estimates.

The results indicate a significant negative correlation between IRTs and confidence ratings for the Short-delay condition (M = -0.17, SD = 0.425) (t(70) = -3.50, Cohen’s d = -0.42, *p* < .001, CI 95% [-0.34, -0.09], BF_10_ = 30.5). Thus, the longer it took for participants to retrieve an object, the lower their confidence. For the Long-delay condition, the results were also significant (M = -0.24, SD = 0.521) (t(70) = -2.45, Cohen’s d = -0.29, *p* = .02, 95% CI [-0.60, -0.07], BF_10_ = 2.08). The results indicate no significant difference in the correlation values between delay conditions (t(70) = 0.91, Cohen’s d = 0.11, *p* = .34, 95% CI -0.18, 0.46], BF_01_ = 5.16).

## Discussion

The results from Experiment 2 generally replicated those of Experiment 1. Regarding objective memory performance, as in the first experiment, the quality of the underlying representations of memories associated with contextual information remained relatively intact after a delay. The proportion of correctly recalled objects was greater in the Short-delay condition than in the Long-delay condition, indicating a significant decline in memory accessibility over time. Temporal organization, background clustering, and semantic organization scores were not negatively affected by delay. In fact, temporal organization was numerically lower in the Short-delay condition, where it did not exceed chance level, compared to the Long-delay condition. The subjective measures’ results also mostly replicated Experiment 1’s, with a decline in confidence ratings after delay for both objects and backgrounds. This may be partly due to reduced retrieval strength over time^34^, which influences subjective confidence ratings, even when the underlying memory representations remain relatively stable. Furthermore, here, too, participants showed an ability to subjectively distinguish between accurate and inaccurate objects and sources in the Short and Long-delay conditions.

A few results were not replicated in Experiment 2. First, the difference in confidence ratings between correct source discrimination in the Short and Long-delay conditions was insignificant (though descriptively in the same direction as in Experiment 1). Second, there was no significant difference in the number of errors between delay conditions.

Regarding the IRT results, a significant difference was found between the delay conditions: participants recalled objects faster in the Short-delay condition than in the Long-delay one. This result was in line with our hypothesis that the effort of retrieval increases over time, likely due to reduced accessibility to the representations (even when the quality of the retrieved representations does not change)^10,36^. In contrast to our hypothesis, however, no significant difference was found between IRTs for correct versus intrusions recall.

Additionally, the longer the IRTs, the lower the participants’ confidence ratings. These results align with research in the field of meta-cognition that found that participants rely on retrieval fluency as a cue for making meta-cognition judgments; hence, retrieval times were longer for lower confidence judgments^25,26^. No difference was found between the two delay conditions with respect to the correlations between IRTs and confidence ratings. This might indicate that retrieval fluency does not vary as a function of delay.

### General discussion

The current study focuses on a fundamental question regarding memories associated with contextual information, which has received scant attention: What is the subjective experience accompanying forgetting of such memories? Namely, how does delay-dependent forgetting affect subjective measures of memory, specifically for memories associated with contextual information, whose representations’ nature has been shown relatively stable over time? We hypothesized, and found, that subjective measures of memories will decline over time, even for items that are correctly retrieved and whose representations’ quality remains relatively stable.

We first discuss the pattern of findings for objective memory measures. In line with previous studies^7–9,11^, we found evidence for minimal or no loss of the qualitative information of memories associated with contextual information after delay (and in some cases even an improvement in certain contextual measures). We found reduced accessibility over time, manifested in fewer correctly recalled items in the Long-delay condition than in the Short-delay one. However, given that a participant correctly recalled an item, the nature of its underlying representation was relatively similar in both delay conditions, as we hypothesized.

The results show that TCE, the phenomenon whereby participants tend to successively recall items from close serial positions^5,12,13^, is not hindered by delay-dependent forgetting. This replicated previous findings^7^ and indicates that the role of temporal context in driving recall is not negatively affected by the passage of time. In addition, our results indicate no difference between background clustering effect in both experiments. The temporal and background organization are retrieval cues that rely on the reinstatement of the encoding context at retrieval. Thus, recalling one item reinstates its context, aiding the retrieval of contextually-related memories^37,38^. These results show that participants use similar cues and retrieval strategies in both delay conditions, and are aligned with previous studies that showed that episodic representations of memories associated with contextual information are either fully reinstated or forgotten entirely after delay^9,10,33^. Finally, and as previously demonstrated^7^, there was no difference in the semantic clustering effects between delay conditions in both free-recall experiments. This suggests that the role of semantic information in guiding retrieval is unaffected by delay-dependent forgetting. Since semantic organization is not tied to the contextual details of the memory, a delay-dependent effect on it was not expected.

In contrast to previous results^7,10^, we found a significant difference in the accuracy of source discrimination between delay conditions. This result also contrasts the pattern of results regarding the background clustering measure, which indicated that the role of source information in triggering recall is not detrimentally affected by delay. One possible interpretation of this contradictory pattern is that in the current study, the background source was an inherent part of the item. Hence, the representation of the word/object alongside its background can be conceptualized as a paired-associate, or even a unitized representation^39–42^. That source discrimination was very high in both experiments might be in line with this idea. Given this interpretation, the decline in source discrimination, in fact, reflects a decline in the accessibility of the memory representation, which includes both the word/object and the background (rather than a decline in the quality of the memory representation). Background clustering, in this case, can be regarded as reflecting organization of memories according to item- (rather than context-) information, similarly to semantic clustering.

Our conclusions regarding the relative stability in the quality of memory representations only pertain to correctly recalled items. Similar conclusions cannot be drawn about missing items—namely, items which participants did not overtly recall, even though they may have retained some item and/or source information regarding them. For such items, the underlying memory representations might not have remained stable over time.

Most importantly, our results provide novel evidence for a discrepancy between objective and subjective measures of memories associated with contextual information. While the quality of the memory representation hardly changes over time, participants’ confidence in their memory declines. A possible interpretation of these results is that as delay increases, retrieval strength diminishes^34,43^ and participants’ ability to access information becomes less reliable. This, in turn, leads to slower and less confident retrieval. Although the quality of underlying memory representations remains relatively stable, the reduced ease of retrieval diminishes the accuracy of subjective confidence ratings. The relationship between retrieval strength and retrieval fluency further clarifies the discrepancy between objective and subjective memory measures over time. Retrieval strength and retrieval fluency are two related, but distinct, phenomena: retrieval strength represents the cognitive accessibility of a memory trace^34,43^, while retrieval fluency refers to the subjective ease of recalling that memory^25^. Retrieval strength is associated with the ease with which memories can be accessed^34,43^ and plays a critical role in shaping subjective confidence. As retrieval strength decreases over time^34^, the ability to access information becomes less reliable, which naturally leads to lower confidence ratings. This process is intricately linked to ease of retrieval (or retrieval fluency, which is the subjective experience of how easily information comes to mind^25^^44^. When retrieval becomes slower and more effortful due to reduced retrieval strength^34,43^, retrieval fluency declines, which results in lower confidence ratings and potentially more variability in subjective accuracy. Thus, while the underlying memory representation may remain relatively stable, the experience of retrieval fluency—and the resulting confidence—diminishes over time, leading to a reduced alignment between objective accuracy and subjective confidence.

A possible explanation for the change in objective-subjective correspondence over time can be derived from the cue-utilization theory. According to this theory, meta-cognitive judgments, such as confidence ratings, are inferential and based on two types of cues: experience-based and theory-based^35,45,46^. Experience-based cues are devoid of declarative content and instead rely on subjective feelings and heuristics, such as the ease with which the items are processed during encoding. Theory-based cues, on the other hand, refer to analytic inferences derived from declarative content, such as beliefs or prior theories regarding memory skills and abilities^35,46^. The accuracy of meta-cognitive judgments is a function of the validity of these cues. Hence, invalid cues, such as biased heuristics cues, may lead to a discrepancy between subjective and objective measures of memory^21^. In the case of the current study, theory-based cues may not reflect that some aspects of memory show little or no forgetting^23^, and consequently, participants might have rated their confidence lower even when their memories remained unchanged over time^47^. This suggests that participants may have assumed that memory naturally declines over time, which could have influenced their confidence ratings.

Experience-based cues, such as retrieval fluency, seem to have played a role in both Short- and Long-delay conditions. This is supported by the IRT results, which showed that shorter retrieval times were associated with higher confidence in both conditions. This indicates that participants effectively used retrieval fluency as a cue for confidence judgments. Additionally, the negative correlation between IRTs and confidence ratings underscores the relationship between retrieval ease and subjective confidence^44,48^. These findings align with previous research showing longer response times for lower meta-cognitive judgments across various domains^26–29,49–56^.

Here we investigated the relationship between objective and subjective memory measures after a delay of 24 h. An interesting question for future research is whether the accuracy-confidence relation changes with various retention intervals. A recent meta-analysis found that different timescales of study-test intervals (1 min to 1 day, 12 h to 7 days, and beyond seven days) show different forgetting patterns^57^. According to this study, memories undergo four different phases over time, each characterized by its distinct forgetting function. Therefore, the relation between objective and subjective memory performance may change depending on the timescale.

One potential limitation of our study is the influence of non-criterial recollection, which refers to retrieving episodic details that are not directly relevant to the memory task but may still affect participants’ confidence and retrieval decisions^58^. Prior research has shown that non-criterial recollection can influence memory judgments by introducing additional details that are not required for the task but still contribute to subjective assessments of memory^59^. In our study, it is possible that participants retrieved incidental details that were not part of the primary retrieval criteria, which could have affected confidence ratings and the overall recall pattern. While our design primarily focused on criterial recollection, future research could further examine the role of non-criterial recalled by incorporating methodological approaches that distinguish between retrieval of task-relevant and incidental details. Such approaches may potentially show differences in the role of non-criterial recollection over time.

An additional limitation of the current study is that the requirement for multiple judgments after each recalled item. This may have introduced some degree of output interference, potentially reducing the likelihood of retrieving weaker memories. While this is an inherent challenge in subjective memory assessment studies, future research may explore alternative designs to mitigate this effect.

Last, we acknowledge that free recall is a more demanding test than recognition and primarily captures the strongest memories. This limitation could underrepresent weaker memories that might still be accessible through recognition. However, free recall was chosen deliberately as it provides a more stringent test of memory retrieval, allowing us to examine the accessibility of contextual memories without external cues. Free recall can be a sensitive measure of memory organization and retrieval strategies, revealing qualitative aspects of memory that might not be as evident in recognition tasks. In summary, the current study provides evidence for a discrepancy between objective measures and subjective experiences of forgetting memories associated with contextual information over time. Despite relative stability in the nature of the memory representations over time, there was a significant decline in subjective confidence.

## Methods

### Experiment 1

#### Participants

The sample size was determined by conducting a power analysis using Gpower 3.1.9.4 for within-subjects t-tests, for the effect of delay condition on recall accuracy. The estimated effect (Cohen’s d) size was 0.4. Here and in all subsequent power calculations, an alpha of 0.05 and an a priori power of 0. 90 were used. This yielded a sample size of 55 participants.

All participants were from Ben-Gurion University and took part in this experiment in return for course credit or a monetary award of 25NIS (approximately 7 USD$). Demographic information on gender and age was collected by the Experimental system of the Department of Psychology at Ben Gurion University. Only participants who are native Hebrew speakers with no diagnosis of attention disorders or other neurological deficits were included in the experiment. The research was reviewed and approved by the Research Committee on Human Issues of the Department of Psychology, Ben-Gurion University of the Negev, Israel. Participants read and signed informed consent forms indicating their willingness to participate in the study. In addition, all methods were performed in accordance with relevant guidelines and regulations.

During the data collection process, it became apparent that data from a substantial number of participants would be inadequate for calculating certain measures of interest (a comprehensive description of these measures can be found below). We, therefore, continued collecting data beyond the required sample size to account for potential exclusions, ultimately reaching a total of 134 participants (mean age = 26.14 SD = 3.86). This approach was taken to ensure that we would have enough participants with adequate data even after any necessary exclusions.

A total of 58 participants were excluded from the analysis for the following reasons: failure to produce correct recalls in the Long-delay condition (*n* = 32), incalculable temporal factors in the Long-delay condition, failure to recall in the Long-delay condition (*n* = 7), uniform confidence reporting in both delay conditions (*n* = 15). As a result, the final analyses of Experiment 1 contained 76 participants.

The exclusion rate observed in Experiment 1is relatively high, though within the range of that reported in online experiments^60,61^. Indeed, the literature on online experimentation consistently reports high dropout or noncompletion rates. Exclusion rates of over 30% were reported in 20% of online experiments in a recent review (Rodd), and some have reported exclusion rates of over 50%^61^. Longer experiments, like the current one, are especially vulnerable to dropout due to a decline in attention and motivation over time^62^. Importantly, several factors substantially mitigate any concerns regarding the validity of our findings due to the high exclusion rate.

First, our exclusion criteria were determined a priori based on established performance thresholds, and were applied uniformly across participants to ensure data validity rather than to optimize outcomes. Importantly, these criteria are consistent with standard practices for maintaining data integrity in remote cognitive research^60^. In such research, exclusion is likely not reflective of extreme variations in cognitive performance, but rather is the result of susceptibility to attentional lapses or multitasking due to increased environmental distractions^60,62,63^. For instance, Elliott et al.^64^ excluded participants whose recall performance fell below three standard deviations from the mean, arguing that such extreme scores likely reflect lapses of attention or noncompliance rather than genuine memory variability. This approach is conceptually similar to our exclusion criterion, which aimed to preserve the validity of the analyzed data while minimizing noise introduced by uninformative trials.

Second, exclusion of participants’ data is of most concern in studies applying between-subject designs, where dropout may be systematically biased (e.g., greater in one the experimental groups). This is not a concern in within-subject designs such as that applied in the current study. Thus, it is highly unlikely that effects of the delay manipulation were distorted by data exclusion^60^.

Finally, Experiment 2, which implemented improved materials and procedures, yielded substantially lower exclusion rates while reproducing the main findings—demonstrating that the observed effects are robust and not dependent on selective sampling.

#### Materials

##### Words lists

The study included two lists of 32 Hebrew words each, including nouns, verbs, and adjectives. To reduce interference, each list was from a different semantic category: living (e.g., “elephant”) or non-living (e.g., “book”). Assignment of the list category (living/non-living) to condition (Short-delay/Long-delay) was counterbalanced across participants: for half of the participants, the living category was presented in the Short-delay condition, the non-living category in the Long-delay condition, and the reverse for the other half.

The words were selected from a Hebrew pool of free association norms containing 800 words^65^ and were three to nine letters long. To effectively measure semantic organization, we manipulated the semantic distance between words within each list. The semantic distance between word pairs was measured by pathway length and derived from the semantic network constructed by Kenett et al.^66^. In this model, a distance of 1 signifies high semantic similarity, and a distance of 4 indicates lower semantic similarity. The lists were constructed such that items from a 1-distance pair did not appear adjacent to one another, and no adjoining pair had a high similarity. For each list (each semantic category), five different ordering schemes of the words within the list were created, which adhere to the conditions stated above. Each participant was randomly assigned to one of these ordering schemes.

##### Pictures for the memory background task

Each word appeared in the middle of the screen, superimposed on a background picture of a forest or a sea. Both pictures were taken from the Gamoran et al.^7^ study.

#### Procedure

##### Practice phase

**Fig. 7:**
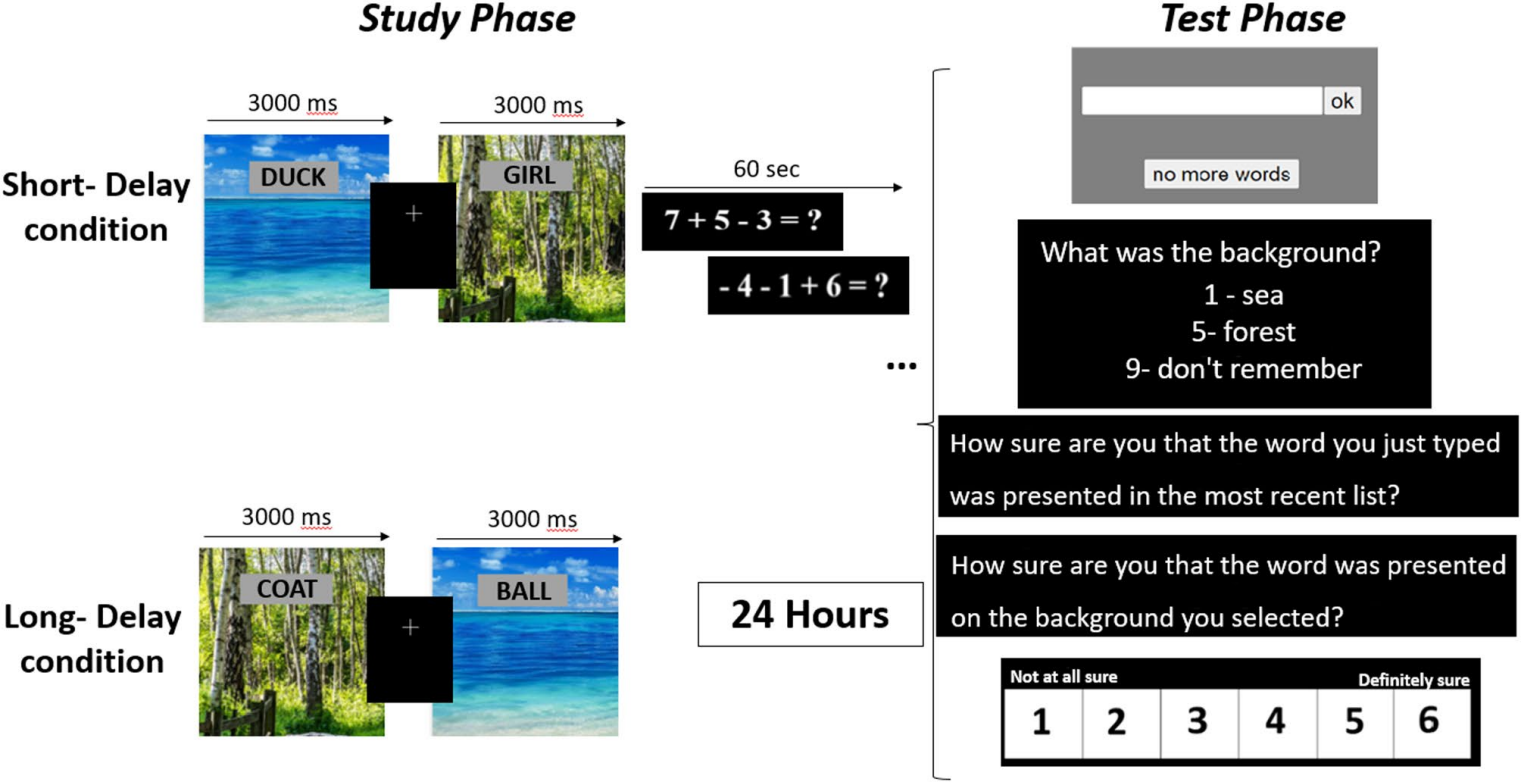
Illustration of Experiment 1 sequence consisting of both delay conditions. A study phase of the Short-delay condition in which a single 32-word list was presented, followed by a distraction task of 6000ms, and a free-recall test for this list. Then, a study phase of a new 32-word list, a delay of 24 h, and a recall test for the most recently presented list.

The method of this Experiment is primarily based on that used by Gamoran et al.^7^ and is presented in Fig. 7. This study employed a within-subjects design, where participants completed both delay conditions: Short-delay first, followed by Long-delay. Before the first study phase, each participant underwent a practice phase that included one fixed list of 8 countries’ names with a background of either sea or forest in random order. Each word appeared for 3000 ms, followed by a fixation cross for 1000 ms. After presenting one list, subjects completed a distraction task in which they were asked to solve simple mathematical problems for 30 s (e.g., “2 + 4 + 9”). They received feedback for correct answers and ☹ feedback for incorrect ones. Following the distraction task, participants completed a free recall test for the countries’ names and a recognition test for the backgrounds. This procedure ensured that the information transitioned from short-term to long-term memory before testing, while minimizing rehearsal and interference. Using a non-verbal distraction task is consistent with previous studies aiming to prevent verbal interference during the retention interval^7^.

##### Study phase

Like the practice phase, each word appeared with a background picture of a sea or a forest (each background picture was paired with half of the words). The stimulus presentation duration was the same on the practice list (each word appeared for 3000 ms, followed by a fixation cross for 1000 ms). Participants were asked to try to remember the background picture with the word. Following the study phase of the Short-delay condition, participants completed the distraction task for 60 s. In the Long-delay condition, the test phase was conducted a day later.

##### Test phase

First, participants were asked to type all the remembered words, one at a time. After each word was typed, another screen was displayed, asking about the background that appeared with the word in the study phase. Participants were asked to respond ‘1’ for ‘sea’, ‘5’ for ‘forest’, or ‘9’ for ‘I do not remember’. Following the background question, participants were asked to rate their confidence regarding the correctness of the word (“How sure are you that this word appeared in the list?“) and the background (“How sure are you that this was the background of the word?“) on a scale of 1 (low confidence) to 6 (high confidence). Participants were given a maximum of two minutes to complete the word recall phase.

#### Analysis

##### General

All data were processed with in-house Matlab scripts (The Mathworks, Natick, MA, USA) and R scripts^67^. The main packages used included *lme4* for mixed-effects models, *BayesFactor* for Bayesian t-tests and ANOVAs, *emmeans* for post-hoc comparisons, and *tidyverse* and *ggpubr* for collection for data preprocessing and visualization.

Statistical analyses, including Bayesian analyses, were performed with JASP version 0.9 and with R version 4.3.0. Data preprocessing included extracting participant responses on the test phase, running them through a spellchecker, correcting misspelled answers, using the hunspell package^68^. When correcting misspelled answers, we accepted answers that included a typo of one letter.

##### Temporal organization

This term refers to the measure of the Temporal Contiguity Effect (TCE, aka “Lag Recency Effect”). The TCE reflects the individual’s tendency to successively recall two items studied in close temporal proximity. A common interpretation of this effect is that it is driven by the gradual evolution of temporal context, such that neighbouring items share similar contextual information, making them more likely to be recalled together^12^. However, it is important to acknowledge that this is one of several possible explanations for the TCE. Alternative models suggest that contiguity effects may arise from mechanisms that do not necessarily require item-level changes in temporal context^13,69^. For instance, Farrell^69^ proposes that episodic memory is structured hierarchically, with recall sequences influenced by both fine-grained and coarse-grained temporal clustering, rather than solely by a gradually shifting temporal context.

To measure this effect, we calculated a Temporal Factor Score for each participant that reflects the utilization of temporal context^8^. This is a single value per participant and per delay condition (Short-delay, Long-delay) that reflects participants’ tendency to recall two words that neighboured at study successively. The score is calculated on so-called *lags*, referring to -the distance between the study serial positions of the two successively recalled words. For each word recalled, all the absolute lags of all possible transitions to that word were calculated, and the absolute lag of the actual transition was also calculated. Each of those transitions was given a Spearman’s rank based on the absolute lag (with the lowest lag given the highest rank). Next, each transition received a Temporal Factor Score between 0 and 1 and based on the following equation: $\:\frac{R-1}{N-1}$ when R is the rank of the actual transition made, and N is the number of possible transitions that could have been made. For each participant, the transitions’ scores were then averaged to yield two scores: one for Short-delay and the second for Long-delay with a value between 0 and 1. A score of 0.5 indicates that chance level temporal organization. A Temporal Factor score of 1 indicates that all the transitions received the highest possible score regarding the temporal organization.

##### Semantic clustering

This term refers to participants’ tendency to recall two items that are related semantically successively. To measure semantic clustering, a similar calculation was performed as that used for Temporal Factor Scores. Each transition between two recalled words received a Semantic Factor Score between 0 and 1 based on the following equation: $\:\frac{R-1}{N-1}$ when N refers to the number of all possible transitions that could have been made between two words in the list, and R refers to the rank of the actual transition in terms of semantic distance between the two words recalled. For each participant and each condition, a Semantic Factor Score was calculated and received a value between 0 and 1, with a score of 0.5 signifying chance level semantic organization^8^.

##### Source discrimination

To examine participants’ source discrimination, namely that which appeared with the word at study, we calculated the proportions of correct responses out of correct and incorrect responses, excluding responses of ‘I do not remember’. Therefore, the chance level was 0.5.

##### Background organization

To examine whether the background picture influenced recall organization, we examined *Background Clustering.* This measure is similar in its rationale to the temporal and semantic organization measures. It is calculated by dividing the number of times a participant successively recalled words that had originally appeared on the same background during the study by the total number of successively recalled words. The background clustering scores were compared to a baseline score, which is the average background clustering score across 500 recall sequences of the same words as those recalled by the subject but shuffled in random order for each list. The baseline score provides a measure of the expected clustering purely based on temporal contiguity. The average baseline score was 0.466, which corresponds to approximately 11/23, representing the odds of recalling another item with the same background immediately after one item has been recalled. To dissociate the effects of temporal and memory background clustering, we calculated a baseline score for each participant in each delay condition, which provides us with a measure of the expected recall sequence on the basis of temporal contiguity alone. The memory background clustering score can vary from 0 to 1, with the baseline score indicating chance level clustering^38^.

We applied a specific exclusion criterion for the analyses of memory organization measures: participants were required to have produced at least two consecutive correct recalls within the same list during the memory test. Participants who did not meet this criterion in either delay condition were excluded from the analyses altogether. This procedure ensured that organizational scores were calculated only for participants with sufficient valid recall sequences, maintaining a rigorous and comparable standard across delay conditions.

## Experiment 2

In the current experiment, we expected to replicate the findings of Experiment 1. In addition, we expected to find effects of both delay and confidence level on IRTs: items that are harder to retrieve following a delay were expected to have longer IRTs and to be given lower subjective ratings^51^. Thus, delay was expected to affect the subjective experience of forgetting^35^, such that items which are harder to retrieve, following a Long-delay, would be associated with lower confidence ratings, even when the quality of their mnemonic representations is relatively resistant to delay.

### Participants

As in Experiment 1, sample size determination was based on an a priori power analysis conducted with GPower 3.1.9.4, targeting a within-subjects t-test comparing recall accuracy across delay conditions. The anticipated effect size was d = 0.4, with α = 0.05 and desired power set to 0.90, resulting in a target sample size of 55 participants.

During the data collection process, it became apparent that a substantial number of participants would not yield analyzable data for several key measures of interest (see below for full exclusion criteria). To account for this, data collection continued beyond the initially determined sample size, ultimately reaching a total of 107 participants (mean age = 26.06, SD = 2.92). A total of 20 participants were excluded from the analyses for the following reasons: failure to produce correct recalls in the Long-delay condition (*n* = 4), incalculable temporal factors in the Long-delay condition (*n* = 3), failure to recall in the Short-delay condition (*n* = 2) or the Long-delay condition (*n* = 2), uniform confidence rating in both delay conditions (*n* = 9). The final analyses of Experiment 2 were therefore based on data from 87 participants.

All participants were from Ben-Gurion University and took part in this experiment in return for course credit or a monetary award of 25NIS (approximately 7 USD$). Demographic information, including age and gender, was collected using the Department of Psychology’s Experimental system. Only native Hebrew speakers with no reported neurological conditions or attention-related diagnoses were eligible to participate.

### Materials

#### Line drawings depicting objects

We constructed two lists of 44 line drawings depicting objects. The line drawing objects were selected from Snodgrass and Vanderwart^29^. In the same way as in Experiment 1, semantic distances were calculated between the words corresponding to the names of the objects. Four different ordering schemes of the objects were created, and each participant was randomly assigned to one of these schemes.

#### Pictures for the memory background task

Each line-drawing appeared in the middle of the screen with a forest or sea background. Both pictures were taken from Gamoran et al.^7^ study.

### Procedure

**Fig. 8:**
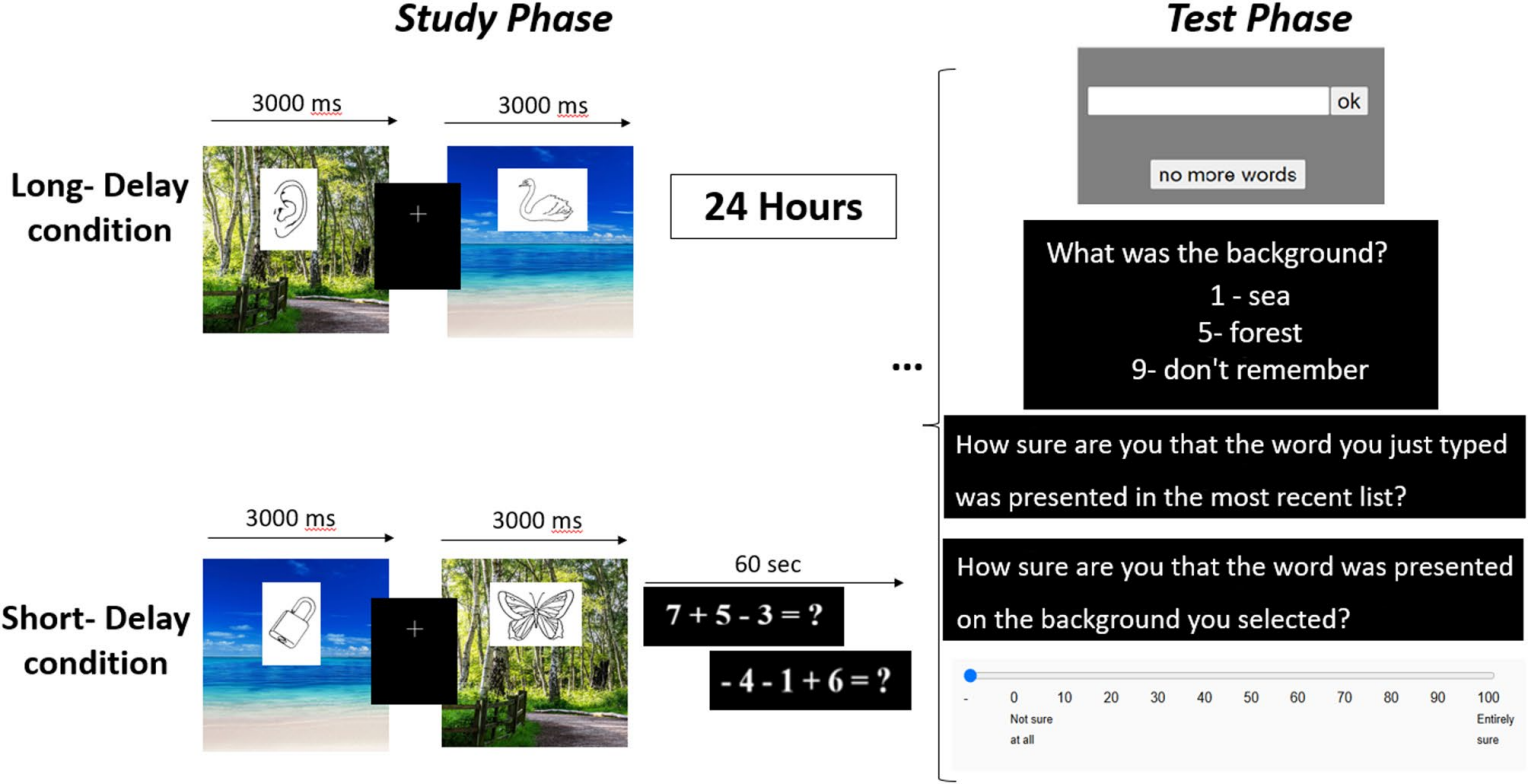
Illustration of Experiment 2 sequence consisting of both delay conditions. A study phase of the Long-delay condition in which a single 44-line drawing objects list was presented with a delay of 24 h and a free-recall test for this list. This was followed by a study phase of a new 44-line drawing objects list with a distraction task of 6000ms, and a free-recall test for the most recently presented list.

The method of this Experiment is presented in Fig. 8. The procedure followed that of Experiment 1 with the following exceptions. First, we used line-drawings rather than words as stimuli. Second, the Long-delay condition preceded the Short-delay one. Thus, participants were presented with the first list of line drawings depicting objects and completed the memory test after a 24 hours’ delay. Following the recall phase, a new list was presented for the Short-delay condition, followed by a 1-minute math problem distraction task. The test phase for the Short-delay condition was performed immediately after the distraction task. Third, we replaced the 1–6 confidence rating scale with a continuous scale from 0% to 100%, as this scale offers a more intuitive approach to rating. All other methodological details were identical to those of Experiment 1, including stimuli presentation durations and recall duration.

### Analysis

The same analyses were done as in Experiment 1, with the addition of an examination of IRTs. IRTs for each recall were measured from when the text box prompting for recall appeared until the participant started typing the name of the remembered object.

Second, as in Experiment 1, we ran participants’ recall through a spellchecker and accepted a few misspelled answers along the same criteria as we did for Experiment 1. In addition, if the line-drawing was ambiguous, we accepted more than one option (e.g., “lettuce” and “cabbage”), as well as both single and plural forms (e.g., both “ear” and “ears”). We also accepted cases in which the correct word depicting the object appeared with an adjective (e.g., “sewing thread” instead of “thread”).

## Supplementary Information

Below is the link to the electronic supplementary material.

Supplementary Material 1

## Data Availability

https://osf.io/2s5j9/?view_only=6df9aa91fac64a5ebd7316cc41ae9313.

## References

[1] Wixted, J. T. The psychology and neuroscience of forgetting. *Annu. Rev. Psychol.* **55**, 235–269 (2004). 14744216 10.1146/annurev.psych.55.090902.141555

[2] McGeoch, J. A. Forgetting and the law of disuse. *Psychol. Rev.* **39**, 352 (1932).

[3] Underwood, B. J. Interference and forgetting. *Psychol. Rev.* **64**, 49–60 (1957). 13408394 10.1037/h0044616

[4] Sadeh, T., Ozubko, J. D., Winocur, G. & Moscovitch, M. How we forget May depend on how we remember. *Trends Cogn. Sci.* **18**, 26–36 (2014). 24246135 10.1016/j.tics.2013.10.008

[5] Sadeh, T., Ozubko, J. D., Winocur, G. & Moscovitch, M. Forgetting patterns differentiate between two forms of memory representation. *Psychol. Sci.* **27**, 810–820 (2016). 27154552 10.1177/0956797616638307

[6] Yonelinas, A. P. The nature of recollection and familiarity: A review of 30 years of research. *J. Mem. Lang.* **46**, 441–517 (2002).

[7] Gamoran, A., Greenwald-Levin, M., Siton, S., Halunga, D. & Sadeh, T. It’s about time: Delay-dependent forgetting of item- and contextual-information. *Cognition* **205**, 104437 (2020). 32861981 10.1016/j.cognition.2020.104437

[8] Sederberg, B., Miller, P. F., Howard, J. W., Kahana, J. & M. & M. The Temporal contiguity effect predicts episodic memory performance. *Mem. Cognit*. **38**, 689–699 (2010). 20852233 10.3758/MC.38.6.689

[9] Berens, S. C., Richards, B. A. & Horner, A. J. Dissociating memory accessibility and precision in forgetting. *Nat. Hum. Behav.* **4**, 866–877 (2020). 32514041 10.1038/s41562-020-0888-8

[10] Joensen, B. H., Gaskell, M. G. & Horner, A. J. United we fall: all-or-none forgetting of complex episodic events. 10.1037/xge0000648 PMC6951107 31305093

[11] Andermane, N., Joensen, B. H. & Horner, A. J. Forgetting across a hierarchy of episodic representations. *Curr. Opin. Neurobiol.* **67**, 50–57 (2021). 32882596 10.1016/j.conb.2020.08.004

[12] Howard, M. W. & Kahana, M. J. Contextual variability and serial position effects in free recall. *J. Exp. Psychol. Learn. Mem. Cogn.* **25**, 923–941 (1999). 10439501 10.1037//0278-7393.25.4.923

[13] Kahana, M. J. Associative retrieval processes in free recall. *Mem. Cognit*. **24**, 103–109 (1996). 8822162 10.3758/bf03197276

[14] Sadeh, T. & Pertzov, Y. Scale-invariant characteristics of forgetting: toward a unifying account of hippocampal forgetting across short and long timescales. *J. Cogn. Neurosci.* **32**, 386–402 (2020). 31659923 10.1162/jocn_a_01491

[15] Iii, R., Wixted, H. L. & Desoto, K. A. J. H. The curious complexity between confidence and accuracy in reports from memory. in Memory and Law (eds (eds Nadel, L. & Sinnott-Armstrong, W. P.) 84–117 (Oxford University Press, doi:10.1093/acprof:oso/9780199920754.003.0004. (2012).

[16] Luus, C. A. E. & Wells, G. L. The malleability of eyewitness confidence: Co-witness and perseverance effects. *J. Appl. Psychol.* **79**, 714–723 (1994).

[17] Wells, G. L., Lindsay, R. C. & Ferguson, T. J. Accuracy, confidence, and juror perceptions in eyewitness identification. *J. Appl. Psychol.* **64**, 440 (1979). 489504

[18] Brewer, N. & Wells, G. L. The confidence-accuracy relationship in eyewitness identification: effects of lineup instructions, foil similarity, and target-absent base rates. *J. Exp. Psychol. Appl.* **12**, 11–30 (2006). 16536656 10.1037/1076-898X.12.1.11

[19] Lindsay, D. S., Read, J. D. & Sharma, K. Accuracy and confidence in person identification: the relationship is strong when witnessing conditions vary widely. *Psychol. Sci.* **9**, 215–218 (1998).

[20] Saraiva, R. B. et al. Using metamemory measures and memory tests to estimate eyewitness free recall performance. *Memory* (2019). https://www.tandfonline.com/doi/full/10.1080/09658211.1688835 (2020). 10.1080/09658211.2019.1688835 31699019

[21] Spearing, E. R. & Wade, K. A. Long retention intervals impair the confidence–accuracy relationship for eyewitness recall. *J. Appl. Res. Mem. Cogn.* **11**, 384 (2022).

[22] Odinot, G. & Wolters, G. Repeated recall, retention interval and the accuracy–confidence relation in eyewitness memory. *Appl. Cogn. Psychol.* **20**, 973–985 (2006).

[23] Sekeres, M. J. et al. Recovering and preventing loss of detailed memory: differential rates of forgetting for detail types in episodic memory. *Learn. Mem.* **23**, 72–82 (2016). 26773100 10.1101/lm.039057.115 PMC4749834

[24] Kelly, A., Carroll, M. & Mazzoni, G. Metamemory and reality monitoring. *Appl. Cogn. Psychol.* **16**, 407–428 (2002).

[25] Koriat, A. & Ma’ayan, H. The effects of encoding fluency and retrieval fluency on judgments of learning. *J. Mem. Lang.* **52**, 478–492 (2005).

[26] Son, L. K. & Metcalfe, J. Judgments of learning: evidence for a two-stage process. *Mem. Cognit*. **33**, 1116–1129 (2005). 16496730 10.3758/bf03193217

[27] Reber, P. J., Alvarez, P. & Squire, L. R. Reaction time distributions across normal forgetting: searching for markers of memory consolidation. *Learn. Mem.* **4**, 284–290 (1997). 10456069 10.1101/lm.4.3.284

[28] Rubin, D. C., Hinton, S. & Wenzel, A. The precise time course of retention. *J. Exp. Psychol. Learn. Mem. Cogn.* **25**, 1161–1176 (1999).

[29] Snodgrass, J. G. & Surprenant, A. Effect of retention interval on implicit and explicit memory for pictures. *Bull. Psychon Soc.* **27**, 395–398 (1989).

[30] Dunlosky, J. & Metcalfe, J. *Metacognition* (SAGE, 2008).

[31] Harlow, I. M. & Donaldson, D. I. Source accuracy data reveal the thresholded nature of human episodic memory. *Psychon Bull. Rev.* **20**, 318–325 (2013). 23192370 10.3758/s13423-012-0340-9

[32] Harlow, I. M. & Yonelinas, A. P. Distinguishing between the success and precision of recollection. *Memory* **24**, 114–127 (2016). 25494616 10.1080/09658211.2014.988162 PMC4466092

[33] Balaban, H., Assaf, D., Arad Meir, M. & Luria, R. Different features of real-world objects are represented in a dependent manner in long-term memory. *J. Exp. Psychol. Gen.* **149**, 1275–1293 (2020). 31804123 10.1037/xge0000716

[34] Bjork, R. & Bjork, E. A. New theory of disuse and an old theory of stimulus fluctuation. (1992).

[35] Koriat, A. Monitoring one’s own knowledge during study: A cue-utilization approach to judgments of learning. *J. Exp. Psychol. Gen.* **126**, 349 (1997).

[36] Koriat, A., Bjork, R. A., Sheffer, L. & Bar, S. K. Predicting one’s own forgetting: the role of experience-based and theory-based processes. *J. Exp. Psychol. Gen.* **133**, 643–656 (2004). 15584811 10.1037/0096-3445.133.4.643

[37] Tulving, E. Episodic memory: from mind to brain. *Annu. Rev. Psychol.* **53**, 1–25 (2002). 11752477 10.1146/annurev.psych.53.100901.135114

[38] Polyn, S. M., Norman, K. A. & Kahana, M. J. A context maintenance and retrieval model of organizational processes in free recall. *Psychol. Rev.* **116**, 129–156 (2009). 19159151 10.1037/a0014420 PMC2630591

[39] Sadeh, T., Dang, C., Gat-Lazer, S. & Moscovitch, M. Recalling the firedog: individual differences in associative memory for unitized and nonunitized associations among older adults. *Hippocampus* **30**, 130–142 (2020). 31348573 10.1002/hipo.23142

[40] Diana, R. A., Yonelinas, A. P. & Ranganath, C. The effects of unitization on familiarity-based source memory: testing a behavioral prediction derived from neuroimaging data. *J. Exp. Psychol. Learn. Mem. Cogn.* **34**, 730–740 (2008). 18605864 10.1037/0278-7393.34.4.730 PMC2605011

[41] Quamme, J. R., Yonelinas, A. P. & Norman, K. A. Effect of unitization on associative recognition in amnesia. *Hippocampus* **17**, 192–200 (2007). 17203466 10.1002/hipo.20257

[42] Parks, C. M. & Yonelinas, A. P. The importance of unitization for familiarity-based learning. *J. Exp. Psychol. Learn. Mem. Cogn.* **41**, 881–903 (2015). 25329077 10.1037/xlm0000068 PMC4404176

[43] Bjork, E. L. & Bjork, R. A. Making things hard on yourself, but in a good way: creating desirable difficulties to enhance learning. In:* Psychology and the Real World: Essays Illustrating Fundamental Contributions To Society* (56–64) (USA, (2011).

[44] Kelley, C. M. & Lindsay, D. S. Remembering mistaken for knowing: ease of retrieval as a basis for confidence in answers to general knowledge questions. *J. Mem. Lang.* **32**, 1–24 (1993).

[45] Dunlosky, J., Mueller, M. L. & Thiede, K. W. *Methodology for Investigating Human Metamemory*1 (Oxford University Press, 2015).

[46] Koriat, A. & Metacognition Decision making Processes in Self-monitoring and Self‐regulation. in *The Wiley Blackwell Handbook of Judgment and Decision Making* (eds Keren, G. & Wu, G.) 356–379 (Wiley, 2015). 10.1002/9781118468333.ch12

[47] Buratti, S., Allwood, C. M. & Johansson, M. Stability in the metamemory realism of eyewitness confidence judgments. *Cogn. Process.* **15**, 39–53 (2014). 23812626 10.1007/s10339-013-0576-y

[48] Nelson, T. O. & Metamemory a theoretical framework and new findings. In: *Psychology of Learning and Motivation* 26 125–173 (Elsevier,1990).

[49] Dildine, T. C., Necka, E. A. & Atlas, L. Y. Confidence in subjective pain is predicted by reaction time during decision making. *Sci. Rep.* **10**, 21373 (2020). 33288781 10.1038/s41598-020-77864-8 PMC7721875

[50] Lipton, J. P. On the psychology of eyewitness testimony. *J. Appl. Psychol.* **62**, 90–95 (1977).

[51] Robinson, M. D. & Johnson, J. T. Recall memory, recognition memory, and the eyewitness confidence–accuracy correlation. *J. Appl. Psychol.* **81**, 587–594 (1996).

[52] Robinson, M. D., Johnson, J. T. & Herndon, F. Reaction time and assessments of cognitive effort as predictors of eyewitness memory accuracy and confidence. *J. Appl. Psychol.* **82**, 416–425 (1997). 9190148 10.1037/0021-9010.82.3.416

[53] Stephenson, G. M. Accuracy and confidence in testimony: A critical review and some fresh evidence. In: *Psychology and law: Topics from an international conference* (229–248) (Wiley, 1984).

[54] Stephenson, G. M., Wagner, W. & Brandstatter, H. An experimental study of social performance and delay on the testimonial validity of story recall. *Eur. J. Soc. Psychol.* **13**, 175–191 (1983).

[55] Stephenson, G. M., Clark, N. K. & Wade, G. S. Meetings make evidence? an experimental study of collaborative and individual recall of a simulated police interrogation.

[56] Zylberberg, A., Fetsch, C. R. & Shadlen, M. N. The influence of evidence volatility on choice, reaction time and confidence in a perceptual decision. *eLife*** 5**, e17688 (2016). 10.7554/eLife.17688 PMC5083065 27787198

[57] Radvansky, G. A., Doolen, A. C., Pettijohn, K. A. & Ritchey, M. A new look at memory retention and forgetting. *J. Exp. Psychol. Learn. Mem. Cogn.* **48**, 1698–1723 (2022). 35084927 10.1037/xlm0001110

[58] Yonelinas, A. P. & Jacoby, L. L. Noncriterial recollection: familiarity as Automatic, irrelevant recollection. *Conscious. Cogn.* **5**, 131–141 (1996). 8978527

[59] Parks, C. M. The role of noncriterial recollection in estimating recollection and familiarity. *J. Mem. Lang.* **57**, 81–100 (2007). 18591986 10.1016/j.jml.2007.03.003 PMC2083555

[60] Rodd, J. M. Moving experimental psychology online: how to obtain high quality data when we can’t see our participants. *J. Mem. Lang.* **134**, 104472 (2024).

[61] Uittenhove, K., Jeanneret, S. & Vergauwe, E. From lab-testing to web-testing in cognitive research: who you test is more important than how you test. *J Cogn* **6**, 13 . 10.5334/joc.259 PMC9854315 36721797

[62] Gagné, N. & Franzen, L. How to run behavioural experiments online: best practice suggestions for cognitive psychology and neuroscience. *Swiss Psychol. Open.* **3**, 1 (2023).

[63] Arechar, A. A., Gächter, S. & Molleman, L. Conducting interactive experiments online. *Exp. Econ.* **21**, 99–131 (2018). 29449783 10.1007/s10683-017-9527-2 PMC5807491

[64] Elliott, E. M., Bell, R., Gorin, S., Robinson, N. & Marsh, J. E. Auditory distraction can be studied online! A direct comparison between in-Person and online experimentation. *J. Cogn. Psychol.* **34**, 307–324 (2022).

[65] Rubenstein, O., Anaki, D., Henik, A., Drori, S. & Paran, Y. Hebrew free association norms. *Hebr. Word Norms Hebr.* 17–34 (2005).

[66] Kenett, Y. N., Kenett, D. Y., Ben-Jacob, E. & Faust, M. Global and local features of semantic networks: evidence from the Hebrew mental lexicon. *PLoS ONE*. **6**, e23912 (2011). 21887343 10.1371/journal.pone.0023912 PMC3161081

[67] R: The R project for statistical computing. https://www.r-project.org/

[68] Ooms, J. & details, A. of libhunspell (see A. file) hunspell author. hunspell: High-performance stemmer, Tokenizer, and spell checker. (2024).

[69] Farrell, S. Temporal clustering and sequencing in short-term memory and episodic memory. *Psychol. Rev.* **119**, 223–271 (2012). 22506678 10.1037/a0027371

