# Supplementary material for: The effects of delay on objective memory and on the subjective experience of forgetting
Source: Sci Rep. 2025 Nov 25;15:42002. doi: 10.1038/s41598-025-26057-2 (PMC12647853; doi:10.1038/s41598-025-26057-2)
Supplement: Supplementary file 1 — Supplementary Material 1 [file 41598_2025_26057_MOESM1_ESM.docx]

**Supplementary Materials**

|  | **Short-delay Words** | | **Short-delay Backgrounds** | | **Long-delay Words** | | **Long-delay Backgrounds** | |
| --- | --- | --- | --- | --- | --- | --- | --- | --- |
|  | M | SD | M | SD | M | SD | M | SD |
| **n correct recall** | 7.99 | 2.85 | 6.94 | 2.79 | 4.28 | 2.22 | 3.33 | 2.16 |
| **p correct recall** | 0.94 | 0.11 | 0.82 | 0.19 | 0.88 | 0.18 | 0.68 | 0.24 |
| **n errors** | 0.47 | 0.84 | 0.77 | 0.89 | 0.68 | 1.24 | 0.63 | 0.75 |
| **n don't remember** |  |  | 0.60 | 0.94 |  |  | 1.03 | 1.41 |
| **confidence for correct recall** | 5.65 | 0.53 | 5.23 | 1.15 | 4.67 | 1.00 | 4.37 | 1.54 |
| **confidence for errors** | 4.41 | 1.41 | 3.90 | 1.57 | 3.67 | 1.58 | 3.00 | 1.58 |
| **temporal factor** | 0.57 | 0.15 |  |  | 0.65 | 0.24 |  |  |
| **semantic factor** | 0.56 | 0.14 |  |  | 0.56 | 0.24 |  |  |
| **background factor** | 0.56 | 0.25 |  |  | 0.61 | 0.34 |  |  |

**Table S1.** Experiment 1 data for each variable.

|  | **Short-delay Objects** | | **Short-delay Backgrounds** | | **Long-delay Objects** | | **Long-delay Backgrounds** | |
| --- | --- | --- | --- | --- | --- | --- | --- | --- |
|  | M | SD | M | SD | M | SD | M | SD |
| **n correct recall** | 11.83 | 6.49 | 10.06 | 6.80 | 6.90 | 4.20 | 5.80 | 3.88 |
| **p correct recall** | 0.87 | 0.11 | 0.70 | 0.18 | 0.77 | 0.16 | 0.62 | 0.20 |
| **n errors** | 1.68 | 1.57 | 1.36 | 1.36 | 2.12 | 1.76 | 0.95 | 1.06 |
| **n don't remember** |  |  | 2.30 | 2.00 |  |  | 2.53 | 2.03 |
| **confidence for correct recall** | 86.82 | 17.13 | 83.22 | 20.27 | 79.79 | 16.68 | 77.34 | 21.43 |
| **confidence for errors** | 83.29 | 22.30 | 56.77 | 29.65 | 66.35 | 23.74 | 61.18 | 25.70 |
| **temporal factor** | 0.52 | 0.14 |  |  | 0.57 | 0.21 |  |  |
| **semantic factor** | 0.54 | 0.12 |  |  | 0.56 | 0.18 |  |  |
| **background factor** | 0.56 | 0.19 |  |  | 0.51 | 0.32 |  |  |

**Table S2.** Experiment 2 data for each variable.
